# Cryptic-site binding mechanism of medium-sized Bcl-xL inhibiting compounds elucidated by McMD-based dynamic docking simulations

**DOI:** 10.1038/s41598-021-84488-z

**Published:** 2021-03-03

**Authors:** Gert-Jan Bekker, Ikuo Fukuda, Junichi Higo, Yoshifumi Fukunishi, Narutoshi Kamiya

**Affiliations:** 1 Institute for Protein Research, Osaka University, 3-2 Yamadaoka, Suita, Osaka 565-0871 Japan; 2 Graduate School of Simulation Studies, University of Hyogo, 7-1-28 Minatojima Minami-machi, Chuo-ku, Kobe, Hyogo 650-0047 Japan; 3 Cellular and Molecular Biotechnology Research Institute, National Institute of Advanced Industrial Science and Technology (AIST), 2-3-26, Aomi, Koto-ku, Tokyo, 135-0064 Japan

**Keywords:** Computational biophysics, Computational chemistry, Biophysical chemistry

## Abstract

We have performed multicanonical molecular dynamics (McMD) based dynamic docking simulations to study and compare the binding mechanism between two medium-sized inhibitors (ABT-737 and WEHI-539) that bind to the cryptic site of Bcl-xL, by exhaustively sampling the conformational and configurational space. Cryptic sites are binding pockets that are transiently formed in the apo state or are induced upon ligand binding. Bcl-xL, a pro-survival protein involved in cancer progression, is known to have a cryptic site, whereby the shape of the pocket depends on which ligand is bound to it. Starting from the apo-structure, we have performed two independent McMD-based dynamic docking simulations for each ligand, and were able to obtain near-native complex structures in both cases. In addition, we have also studied their interactions along their respective binding pathways by using path sampling simulations, which showed that the ligands form stable binding configurations via predominantly hydrophobic interactions. Although the protein started from the apo state, both ligands modulated the pocket in different ways, shifting the conformational preference of the sub-pockets of Bcl-xL. We demonstrate that McMD-based dynamic docking is a powerful tool that can be effectively used to study binding mechanisms involving a cryptic site, where ligand binding requires a large conformational change in the protein to occur.

## Introduction

**Figure 1:**
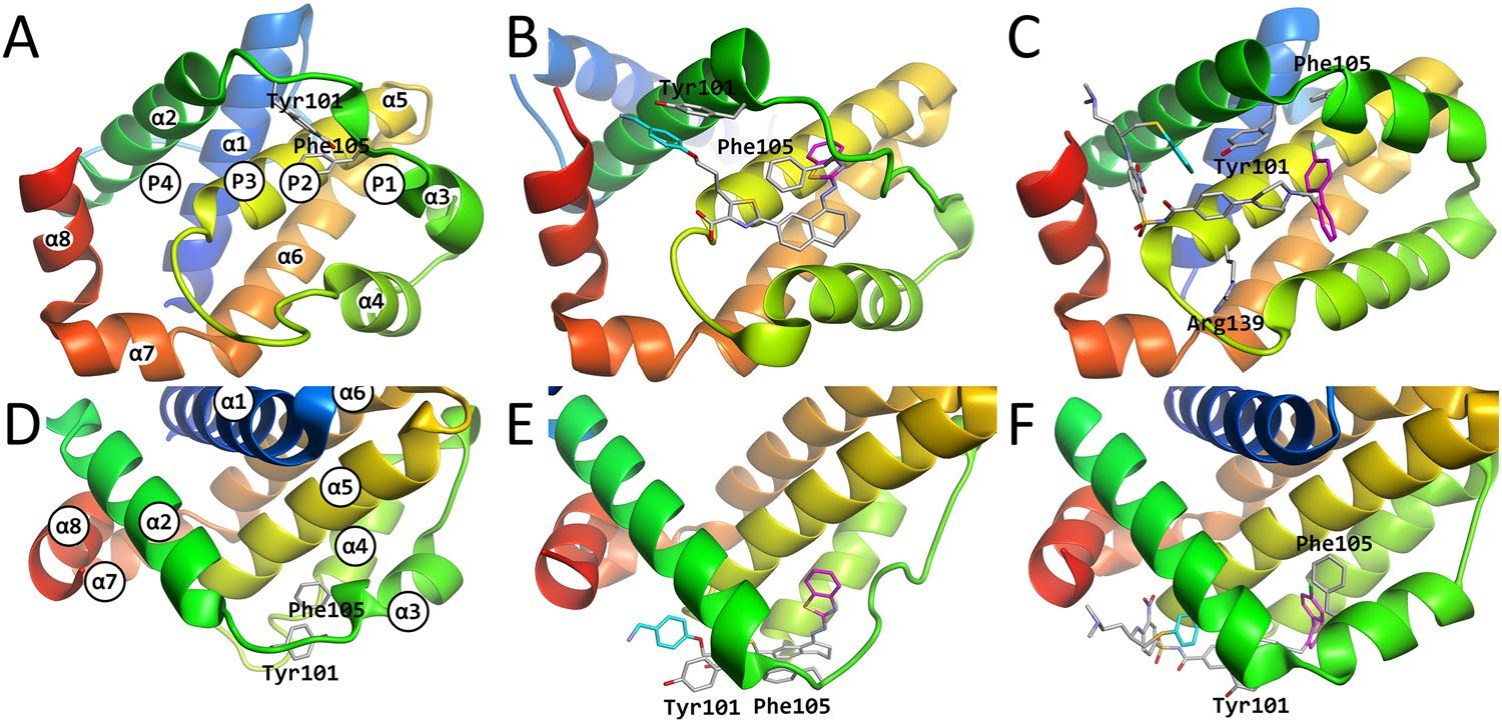
Comparison between the experimental apo structure (**A**/**D**), the WEHI-539-bound structure (**B**/**E**) and the ABT-737-bound structure (**C**/**F**). In panel (**A**), the locations of each of the P1–P4 sites are shown, while in panels (**B**) and (**C**), the locations of each of the L1–L4 sites of the ligands are shown. In panels (**B**/**C**/**E**/**F**) the L2 and L4 sites are colored magenta and cyan, respectively, as in Figs. S9 and S10. The images were produced using Molmil^54^, a WebGL based molecular viewer developed by PDBj^33,34^.

B cell lymphoma 2 (Bcl-2) family proteins play a central role in regulating the apoptotic pathway inside cells^1,2^. Bcl-xL, along with Bcl-2, Bcl-w, Bfl-1, and Mcl-1 are pro-survival proteins part of the Bcl-2 family^3^ that interact with pro-apoptotic proteins such as effector proteins Bax and Bak^4^, upstream initiator proteins within the pathway, or Bcl-2 homology 3 only (BH3-only) proteins such as Bim, Puma and Bad^5,6^. Bcl-xL suppresses these pro-apoptotic proteins by binding to their BH3-motif, inhibiting their apoptotic signal^2^. The BH3-motif has a helical conformation, with a set of hydrophobic residues interacting with the hydrophobic grooves on the surface of the Bcl-2 family, forming a protein–protein complex. Four of these sub-pockets that bind hydrophobic residues from the BH3-motif have been labeled as P1-P4 (Fig. 1A)^4^. Because overexpression of Bcl-xL is one of the hallmarks of cancer^7^, Bcl-xL is considered to be an important drug target, whereby an BH3-mimic inhibitor that binds to the BH3 binding site would be able to prevent Bcl-xL from inhibiting apoptotic signals, halting the growth of cancer cells.

Binding sites that are exposed upon binding to a ligand (i.e., induced-fit) or that appear transiently in an apo form (i.e., population shift or conformational selection) have become a hot topic in drug development and have been named "cryptic sites". Due to their attractiveness for drug discovery, work has gone into has characterizing and predicting such sites^8,9^. The binding site of Bcl-xL is also considered to be such a cryptic site. Bcl-xL in complex with natural/unnatural-proteins/peptides and chemical compounds as well as its apo-form has been well-studied by X-ray crystallography and nuclear magnetic resonance (NMR) spectroscopy^10^. Bcl-xL is an all α-helical protein consisting of eight α-helices (α1-α8 in Fig. 1A,D), and this fold is conserved among Bcl-2 family proteins. The interface of Bcl-xL, which can bind to pro-apoptotic proteins, contains a large hydrophobic groove formed by α2-α4 with the core helix α5^11^. Drugs targeting this groove are rationally designed based on known crystal structures, which has resulted in the discovery of several medium-sized inhibitor molecules with a molecular weight (MW) of > 500 Da^10^. For example, the compound ABT-737 (MW = 813 Da), which was the first BH3-mimic inhibitor developed^12^, binds to the sub-pockets P2 and P4 (Fig. 1C,F) as it interacts with many residues in α2–α5, as well as α8 via mostly hydrophobic contacts in Bcl-xL^13^. Hydrophobic residues of Tyr101 and Phe105 part of the α2–α3 loop exhibit notable structural differences, depending on the bound partner molecule^10^. This wide area of hydrophobic interactions either with or without backbone structure changes in the α2–α4 region makes it hard to capture their dynamic properties due to its cryptic structure, and thus their binding mechanism remains unclear. Another compound known as WEHI-539 (Fig. 1B,E), which was the first Bcl-xL-selective inhibitor developed^14^, forms an ionic interaction with Arg139, and interacts with the side chain of Phe105 via hydrophobic contacts^14^. In particular, displacement of Phe105 can be observed between the apo-state and WEHI-539-bound Bcl-xL.

Dynamic docking using molecular dynamics (MD) simulations can be used to explore binding configurations between receptor proteins and their ligands^15^. We have developed a dynamic docking implementation based on multicanonical molecular dynamics (McMD, see Section S1 for an explanation of the McMD theory)^16^, which we have applied to a number of cases^17–21^. Besides dynamic docking, we have also applied McMD^22,23^ simulations to the conformational sampling of proteins and peptides^24,25^ and the loop structure prediction of an antibody^26^. With McMD, the bias is correlated with the temperature, enabling McMD simulations to adaptively modulate the bias given the density of states. Thus, the potential energy surface functions as a reaction coordinate, which does not depend on any prior knowledge, such as the natively bound complex structure. The canonical ensemble at any given temperature, which is one of the physico-chemically acceptable ensembles, can be generated from the multicanonical ensemble by using a reweighting procedure. The free energy landscape (FEL), which governs the thermodynamic properties of a system, can then be obtained by mapping the reweighted structural ensemble onto a reaction coordinate such as a binding path or onto one or more principal components obtained by Principal Component Analysis (PCA)^24,27^. Analysis of the FEL then uncovers the stable bound complexes as sampled by the McMD simulation.

Here, we perform two exhaustive McMD-based dynamic docking simulations between Bcl-xL and two ligands; ABT-737 (the first BH3-mimic inhibitor) and WEHI-539 (the first Bcl-xL selective inhibitor), starting from apo structure of Bcl-xL. To prevent unfolding of Bcl-xL, while restraining the structure as little as possible, we employed distance restraints on the Bcl-xL structure, defined based upon its apo structure. While several of our previous McMD docking studies restrained the ligand inside a cylindrical region covering both the binding site and the bulk region^18–20^, for our most recent study, we discarded the cylinder and performed an exhaustive search of the configurational space between heat shock protein 90 (Hsp90) and one of its high affinity inhibitors^21^. Even without the cylinder, we successfully predicted the native binding configuration, while also sampling non-native binding configurations spread over the surface of Hsp90. Here, we have also omitted the cylinder, allowing the ligand to sample the full configurational space and enabling us to do a full-domain binding-site search. We will demonstrate that even for such a complex system, where the shape of the pocket not only depends on whether a ligand is bound, but also on the type of ligand that is bound, in addition to the large size of the ligands and the explosive increase in conformational and configurational variability that this brings, we are still able to predict the native bound configuration in agreement with the experimental structures. In addition, we perform path sampling simulations to estimate the affinities and study the binding mechanism of the molecules in closer detail.

## Results

### Dynamic docking

**Figure 2:**
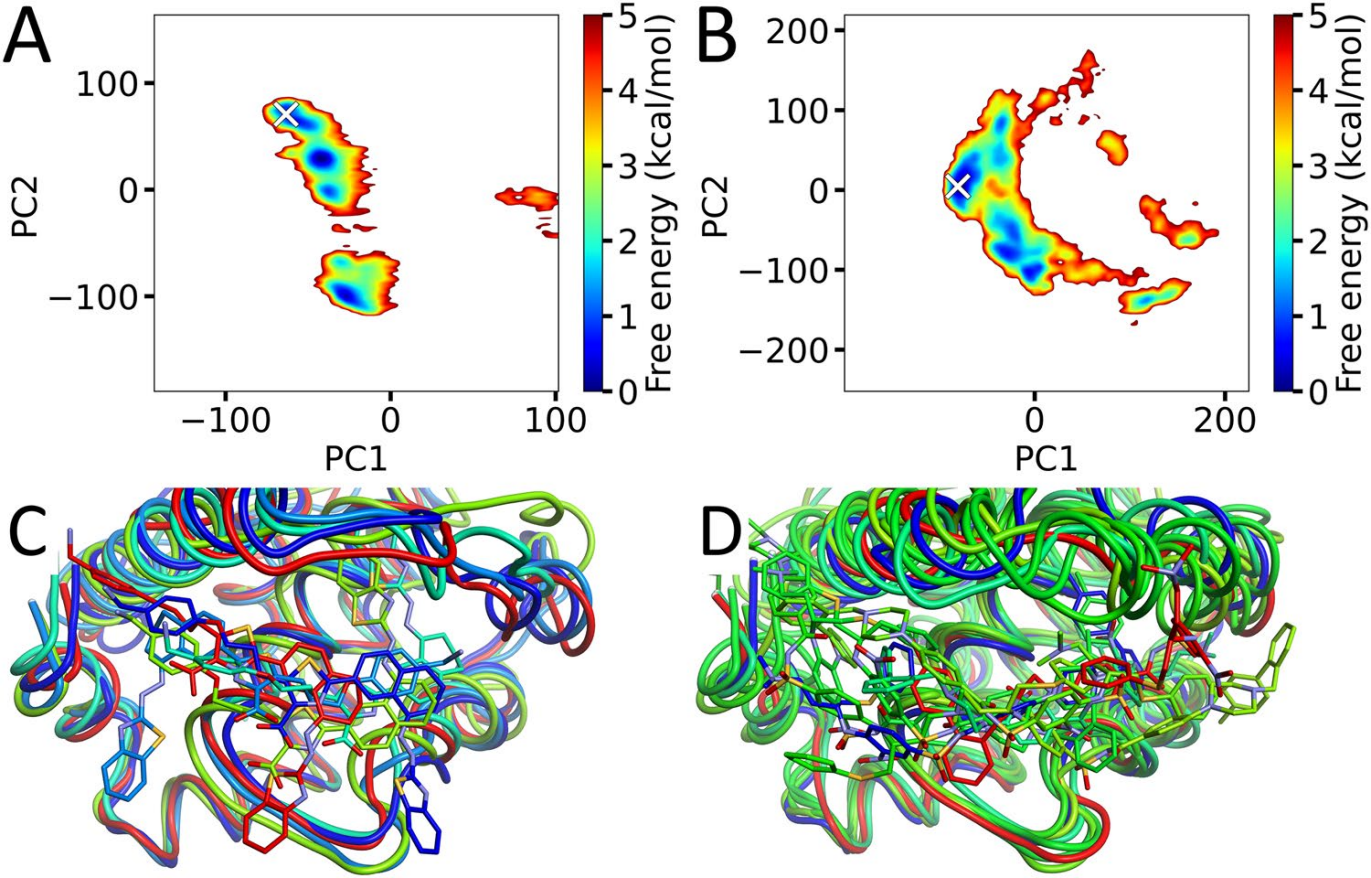
Dynamic docking results. (**A**, **B**) The location of the experimental structure is indicated by the white cross in each panel (**A** for WEHI-539 and **B** for ABT-737) and are located near the global free energy minima. Here, the principal components that have a combined contribution of over 90% have individual contributions of 34.4%, 22.5%, 13.9%, 7.5%, 5.1%, 3.3%, 2.8% and 1.6% for WEHI-539 (PC1–PC8), and 31.2%, 23.7%, 12.1%, 7.9%, 4.7%, 3.6%, 3.0%, 1.9%, 1.7% and 1.5% for ABT-737 (PC1–PC10). (**C**) Structures of ${r}_{k}^{W}$, colored based on their CFE (Table 1). (**D**) Structures of ${r}_{k}^{A}$, colored based on their CFE (Table 2).

After a 750 ns pre-run for each parallel McMD trajectory (N = 32), a production run was executed, with 1 μs per trajectory, producing a multicanonical ensemble consisting of 6.4 × 10^6^ structures for each system. The flat potential energy distribution obtained from the production run is shown in Fig. S1 and Fig. S2 for the WEHI-539 and ABT-737 ligand systems, respectively, with the reweighting distributions (Eq. S5) for *T* at 300 K, 500 K and 700 K. Projecting each of the to 300 K reweighted ensembles onto the first two principal axes obtained via PCA, we obtain independent FELs (see “Methods” section) as shown in Fig. 2A,B. In both cases, the experimental structure is located in the same basin as the global minimum. To study the stable binding configurations in greater detail, we picked representative structures from the multicanonical ensemble for each ligand. Figure 2C,D shows the representative structures **r** of the clusters *k* that have a cluster free energy (CFE) of less than the 1.5 kcal/mol, for the WEHI-539 and ABT-737 system, respectively, with Fig. S3 and S4 showing the locations of these structures **r**_k_ on the FEL. Tables 1 and 2 list several statistics of each structure **r**_k_ for the WEHI-539 (${{\varvec{r}}}_{k}^{W}$) and ABT-737 (${{\varvec{r}}}_{k}^{A}$) system, respectively, where these structures are ranked by the free energy contribution of the corresponding cluster. Fig. S5 and S6 show each of the binding configurations ${{\varvec{r}}}_{k}^{W}$ and ${{\varvec{r}}}_{k}^{A}$, respectively.

**Table 1:** Stable binding configurations obtained for WEHI-539.

	CFE	PC1	PC2	PCA FE	RASA	R(native)-value	RMSD All	RMSD L2	RMSD L4	RMSD center
**r**_1_	0.00	−37.71	31.49	0.27	0.37	0.447	6.91	13.47	1.54	0.58
**r**_2_	0.21	−22.23	−100.5	0.67	0.35	0.178	13.41	18.69	10.45	2.40
**r**_3_	0.48	−59.26	76.63	1.08	0.25	0.752	3.18	2.90	2.52	3.52
**r**_4_	0.95	−49.09	62.65	0.94	0.31	0.491	1.51	1.91	1.29	1.26
**r**_5_	1.50	−34.70	−2.27	1.14	0.42	0.186	9.12	15.75	5.32	5.47
**q**_1_	–	−41.98	28.62	0.12	0.34	0.400	6.89	13.39	1.59	0.30
**q**_2_	–	−21.45	−106.53	1.01	0.32	0.236	13.78	19.31	10.39	1.19
**q**_3_	–	−64.89	71.95	1.13	0.19	0.845	1.39	1.73	0.92	1.12
**q**_4_	–	−53.07	64.31	0.77	0.24	0.552	1.49	2.58	0.88	0.92
Exp	–	−63.45	70.66	1.02	0.18	1.000	0.00	0.00	0.00	0.00

For each representative structure **r**_k_ (from McMD) and each equilibrated structure **q**_k_ (from refinement MD at 300 K), various characteristics are shown. The relative cluster free energy (CFE) value in kcal/mol of the corresponding cluster *k* is shown for the structures **r**_k_. For the structures **r**_k_ and **q**_k_, the first two principal components (PC1-2), the free energy in kcal/mol of the point (PC1, PC2) on the FEL (PCA FE) in Fig. 2A and S3, the fraction of the relative accessible surface area (RASA) of the ligand, the R(native)-value and RMSD (All) in Å with respect to the experimental structure are listed. Finally, the RMSD for different components (L2, L4, center, see Fig. S9) are also listed. For the RMSDs, only the heavy atoms were included. The structures **r**_k_ are ordered by the relative free energy values of their corresponding cluster.

**Table 2:** Stable binding configurations obtained for ABT-737.

	CFE	PC1	PC2	PCA FE	RASA	R(native)-value	RMSD All	RMSD L2	RMSD L4	RMSD center
**r**_1_	0.00	−76.62	5.04	0.01	0.32	0.674	3.87	3.05	4.29	4.77
**r**_2_	0.56	−2.57	−99.05	0.68	0.24	0.172	14.7	12.96	20.20	13.85
**r**_3_	0.69	−82.53	13.01	0.61	0.33	0.844	2.90	4.41	1.36	1.83
**r**_4_	0.71	−75.99	5.33	0.02	0.29	0.570	4.87	2.57	5.52	4.10
**r**_5_	0.78	−29.71	89.62	1.44	0.54	0.228	15.39	25.03	8.98	6.54
**r**_6_	0.89	−20.15	−55.72	1.00	0.35	0.142	13.68	16.35	16.98	8.87
**r**_7_	0.97	−25.17	−82.38	0.81	0.38	0.048	11.84	12.13	11.42	12.29
**r**_8_	1.00	−29.22	−77.88	0.69	0.27	0.101	10.63	9.29	10.59	13.21
**r**_9_	1.50	2.37	−76.72	1.56	0.49	0.128	14.81	9.17	19.19	18.57
**q**_1_	–	−80.08	−1.50	0.27	0.26	0.653	4.03	2.84	4.79	4.03
**q**_2_	–	−3.51	−96.98	0.85	0.26	0.172	14.67	13.74	19.81	13.54
**q**_3_	–	−76.52	14.36	0.20	0.33	0.786	2.69	4.51	1.16	1.31
**q**_4_	–	−76.01	5.03	0.02	0.32	0.563	5.15	3.16	5.74	4.07
**q**_5_	–	−29.90	78.26	1.91	0.60	0.212	15.18	24.46	9.16	6.42
**q**_6_	–	−11.18	−52.06	2.10	0.35	0.151	14.02	15.92	17.58	10.06
**q**_7_	–	5.88	−54.22	2.98	0.52	0.060	12.37	12.15	12.07	12.33
Exp	–	−80.73	4.58	0.14	0.30	1.000	0.00	0.00	0.00	0.00

For each representative structure **r**_k_ (from McMD) and each equilibrated structure **q**_k_ (from refinement MD at 300 K), various characteristics are shown. The relative cluster free energy (CFE) value in kcal/mol of the corresponding cluster *k* is shown for the structures **r**_k_. For the structures **r**_k_ and **q**_k_, the first two principal components (PC1-2), the free energy in kcal/mol of the point (PC1, PC2) on the FEL (PCA FE) in Fig. 2B and S4, the fraction of the relative accessible surface area (RASA) of the ligand, the R(native)-value and RMSD (All) in Å with respect to the experimental structure are listed. Finally, the RMSD for different components (L2, L4, center, see Fig. S10) are also listed. For the RMSDs, only the heavy atoms were included. The structures **r**_k_ are ordered by the relative free energy values of their corresponding cluster.

Most of the top-ranking structures are located in the same basin of the FEL as the experimental structure, showing that our simulations sampled configurations that include the native structure. Looking closely at the statistics however, suggests that the top-ranking configurations (**r**_1_) does not appear to completely match the experimental structures as well as our previous works involving smaller ligands^18,21^, potentially due to the size of the ligands and the large conformational changes to the protein required to allow binding (for WEHI-539). Since the ligands are very large, we have also calculated RMSD values for the different parts (L2, L4 in Figs. S9 and S10) of the ligands to identify the local difference in binding. For WEHI-539, ${{\varvec{r}}}_{1}^{W}$ binds to pocket P4 similar to how the experimental structure binds, but does not bind to P2 in the same way, in particular, L2 is pointing in the opposite direction towards the bulk region. For ${{\varvec{r}}}_{2}^{W}$, both pockets are different, but the center is still close by, suggesting that the orientation is different, which can also be seen Fig. S5. Both ${{\varvec{r}}}_{3}^{W}$ and ${{\varvec{r}}}_{4}^{W}$ are quite similar to the experimental structure (either by RMSD or R-value^19^, see “Methods” section), but don’t rank as high as the ${{\varvec{r}}}_{1}^{W}$ and ${{\varvec{r}}}_{2}^{W}$. Finally, ${{\varvec{r}}}_{5}^{W}$ is a structure similar to ${{\varvec{r}}}_{1}^{W}$, but translated closer to the C-terminal. WEHI-539 thus has a similar structure to the experimental one at rank 3 (${{\varvec{r}}}_{3}^{W}$), while the structure at rank 1 (${{\varvec{r}}}_{1}^{W}$) has L4 bound like the experimental structure, with L2 in a different state, and could potentially be a more easily attainable intermediary structure. For ABT-737, there are more configurations within the 1.5 kcal/mol CFE cutoff, suggesting that entropy of Bcl-xL plays a larger role in the binding of this compound. Configuration ${{\varvec{r}}}_{1}^{A}$ in Fig. S6 is similar to the experimental structure, with ${{\varvec{r}}}_{3}^{A}$ being even more similar and ${{\varvec{r}}}_{4}^{A}$ less similar. Considering that not only the sub-pocket RMSDs don’t match, but also the center is off for ${{\varvec{r}}}_{2}^{A}$, its binding site is away from the experimental one. Taken together, the prediction of ABT-737 went rather well with a similar structure to the experimental one at the top rank (${{\varvec{r}}}_{1}^{A}$).

We have also performed canonical MD simulations at 300 K using the structures ${{\varvec{r}}}_{k}$ to refine them (to produce the equilibrated structures ${{\varvec{q}}}_{k}$) and at 400 K (to analyze their relative stability) for the structures with a CFE of less than 1.0 kcal/mol. The statistics for these structures are also included in Tables 1 and 2 for each structure ${{\varvec{q}}}_{k}$ for the WEHI-539 (${{\varvec{q}}}_{k}^{W}$) and ABT-737 (${{\varvec{q}}}_{k}^{A}$) systems, respectively, with the binding configurations for ${{\varvec{r}}}_{k}^{W}$/${{\varvec{q}}}_{k}^{W}$ and ${{\varvec{r}}}_{k}^{A}$/${{\varvec{q}}}_{k}^{A}$ shown in Fig. S5 and S6, respectively. Similarly, Table S1 and S2 list the obtained average R-values over the final 40 ns of the trajectories and their standard deviations for the 300 K and 400 K canonical MD simulations. Comparing these results with the dynamic docking results shows some interesting findings. The ranking of the canonical simulations at 300 K match quite well with the rankings obtained by the McMD docking simulations, albeit that they are all very close. However, larger differences can be observed for the simulations at 400 K. Furthermore, even the most stable complexes score relatively low, especially compared to our previous simulation of an antibody and a peptide-antigen, which fit together very snuggly^20^. For WEHI-539, both ${{\varvec{r}}}_{3}^{W}$ and ${{\varvec{r}}}_{4}^{W}$ seem very stable at 400 K, suggesting that P2 is important for the stability of the complex structure. Similarly, for ABT-737, ${{\varvec{r}}}_{1}^{A}$ and ${{\varvec{r}}}_{4}^{A}$ are among the most stable configurations, and also match the experimental structure for the P2 sub-pocket. Thus, binding of both ligands looks to be mainly mediated by the P2 sub-pocket, with the P4 sub-pocket only having a supporting role. However, the results also seem to suggest that the initial binding is via P4, followed by P2, where P4 with its lower affinity also has a lower specificity. Given that the preferred structure of P2 depends on which ligand is bound and that P2 contributes considerably to the stability of the complex, P2 binding ligands should be quite specific to Bcl-xL.

We also analyzed the conformation of the protein around the binding site. Table 3 lists the RMSD values and Q-values^28,29^ of the region encompassing Leu99 to Asn136, which corresponds to the binding site. The values of the **q**_k_ structures with respect to the apo structure and the respective holo structures were calculated. Overall, the conformations are often in a state that is neither the apo, nor the holo state. For WEHI-539 ${{\varvec{q}}}_{1}^{W}$ and ${{\varvec{q}}}_{2}^{W}$, the conformation is close to the apo state, but with the ligand in a non-native state. Similar for ABT-737, ${{\varvec{q}}}_{6}^{A}$ is in a conformation close the apo structure, but the ligand is also in a non-native state. For ${{\varvec{q}}}_{3}^{W}$, it is closer to the holo state than the apo state (with the molecules bound similar to the experimental structure), while for ${{\varvec{q}}}_{1}^{A}$ the protein is in a conformation closer to the apo state than the holo state, even though the ligand is similar to the experimental structure. With ${{\varvec{q}}}_{3}^{W}$, L2 is embedded inside the pocket deeper than the experimental structure, with Phe105 positioned to pack better with the ligand near L2. The backbone of the loop that Phe105 is part of in ${{\varvec{q}}}_{3}^{W}$ has a different conformation with Leu108 having moved closer to the surface (after the ligand pushed it out) and Arg103 interacting with Asp107. For ${{\varvec{q}}}_{1}^{A}$, Phe105 is actually positioned more similar to the WEHI-539 experimental structure, with the sidechain making hydrophobic interactions with L2 and Phe97.

**Table 3:** Structural comparison of protein around binding site.

	RMSD Cα apo	RMSD Cα holo	RMSD heavy apo	RMSD heavy holo	Q-value apo	Q-value holo
**WEHI-539**						
**q**_1_	1.39	2.83	2.42	4.09	0.905	0.889
**q**_2_	1.45	2.73	2.60	3.56	0.808	0.894
**q**_3_	3.44	2.33	4.52	3.18	0.588	0.771
**q**_4_	4.67	6.16	5.23	6.28	0.570	0.723
**ABT-737**						
**q**_1_	2.57	3.70	3.87	4.68	0.695	0.755
**q**_2_	3.27	4.73	4.34	5.36	0.666	0.682
**q**_3_	2.26	4.53	3.38	5.28	0.797	0.674
**q**_4_	2.73	3.98	3.64	4.42	0.645	0.880
**q**_5_	2.30	3.76	3.48	4.71	0.873	0.842
**q**_6_	1.68	4.85	2.54	5.32	0.840	0.707
**q**_7_	2.08	4.09	3.14	4.86	0.809	0.682

Values were calculated for the residues Leu99 to Asn136, while the other residues’ Cα atoms were used for the superposition. Both WEHI-539 and ABT-747 were compared to the apo structure (PDB ID: 1R2D) and to their holo structures (PDB IDs 3ZLR and 2YXJ, respectively). Both the Cα-only and all-heavy (Leu99-Asn136) RMSD values in Å were calculated, in addition to the Q-values^28,29^ with respect to the apo and respective holo structures (Leu99-Asn136).

### Binding pathway analysis and affinity calculation

For the first stage, we used our pathing algorithm (see Section S2) to generate a set of structures along an estimated binding/unbinding direction given a reference binding configuration from the multicanonical ensemble. For both ligands, we used our pathing algorithm for the top 4 refined configurations (${{\varvec{q}}}_{1}$–${{\varvec{q}}}_{4}$). Table S3 list the structural similarities of the picked structures between neighboring windows of each of the top 4 refined configurations of both ligands. Notably, for the window at λ = 5 Å of ${{\varvec{r}}}_{3}^{W}$, the similarity to the preceding window is relatively low, with a lax cutoff and low number of matching structures (with the picked ones corresponding to a high temperature, data not shown), suggesting that the transition is quite sudden, as only a relatively low number of structures were sampled along the reaction coordinate at that point. Combined with the observations that the probability (i.e. CFE) of ${{\varvec{r}}}_{1}^{W}$ is higher than ${{\varvec{r}}}_{3}^{W}$ (Table 1), while the stability of ${{\varvec{r}}}_{3}^{W}$ is higher based on the canonical MD simulations at 400 K (Table S1), this would suggest that the two structures are connected only via a very narrow pathway along phase-space.

For the second phase, we use path sampling MD simulations starting from the structures picked in the first phase. We performed path sampling along the reaction coordinate λ, which differs for binding configuration and was calculated using our naïve method from each structure ${{\varvec{q}}}_{k}$^18^. Tables S4 to S7 show the sampling characteristics of the simulation for the WEHI-539 ligand, while Tables S8 to S11 show that for the ABT-737 ligand. Similarly, Fig. S7 shows the potential of mean force (PMF) along the reaction coordinate for each configuration using the final 100 ns of the trajectory data for WEHI-539, while Fig. S8 for that of ABT-737. The affinity values for each of the configurations using the final 100 ns of data is summarized in Table 4. For WEHI-539, the strongest binding configurations are ${{\varvec{q}}}_{3}^{W}$ and ${{\varvec{q}}}_{4}^{W}$, while for ABT-737, ${{\varvec{q}}}_{1}^{A}$ and ${{\varvec{q}}}_{2}^{A}$ bind the strongest. This matches quite well with the data obtained from the canonical simulations at 400 K, especially for WEHI-539. These strong binders all have a PMF value (ΔG) of about 20 kcal/mol, with a standardized free energy (${\mathrm{\Delta G}}_{\mathrm{b}}^{0}$) of −17.95 kcal/mol and −15.45 kcal/mol, for ${{\varvec{q}}}_{3}^{W}$ and ${{\varvec{q}}}_{1}^{A}$, respectively.

**Table 4:** Affinities calculated by path sampling simulations.

	ΔG	σ	ε	${\mathrm{\Delta G}}_{\mathrm{b}}^{0}$
**WEHI-539**				
**q**_1_	16.16	0.01	0.02	−11.63
**q**_2_	16.89	0.01	0.02	−12.21
**q**_3_	22.44	0.01	0.02	−17.95
**q**_4_	20.26	0.01	0.02	−16.00
Exp	–	–	–	−12.66
**ABT-737**				
**q**_1_	20.09	0.01	0.02	−15.45
**q**_2_	20.55	0.01	0.02	−16.12
**q**_3_	16.07	0.01	0.02	−11.56
**q**_4_	19.11	0.01	0.02	−14.50
Exp	–	–	–	−12.90

Affinities were taken from the 150 ns to 250 ns range (i.e. omitting the first 150 ns of simulation and only using the final 100 ns) for the WHAM analyses described in Tables S4–S11. The WHAM analyses were executed using the same parameters as the calculations used in the main text (i.e. a Δλ of 0.05 Å, a tolerance of 1e^−8^ and with 1000 bootstraps). ΔG is the average PMF over the final 50 bins (2.5 Å), with σ its standard deviation. ε corresponds to the average error (via bootstrapping) taken over the same range. ${\mathrm{\Delta G}}_{\mathrm{b}}^{0}$ is the standard binding free energy calculated using ΔG and the corresponding sample COMs during the start and end range of the simulation following Eq. S7. The experimental (Exp) affinities (in the ${\mathrm{\Delta G}}_{\mathrm{b}}^{0}$ column) were obtained from Lessene et al^14^.

**Figure 3:**
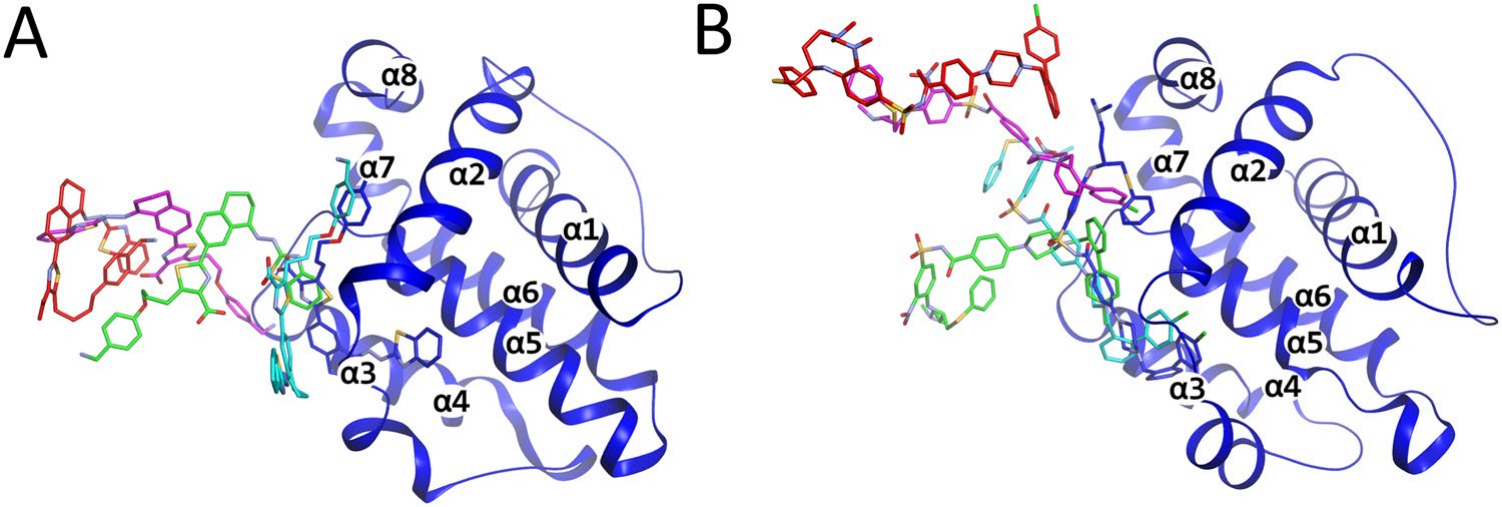
Snapshots taken from path sampling simulations of ${r}_{3}^{W}$ (**A**) and ${r}_{1}^{A}$ (**B**). Representative structures picked at (see “Methods” section) λ = 0, 5, 10, 15 and 20 Å are colored in blue, cyan, green, magenta and red, respectively. The protein is represented as a thin tube model with only the protein structure at λ = 0 Å shown, in addition to the ligand structures as stick models.

Using the methodology that we used previously^19^, we constructed a stitched trajectory of representative structures obtained from the path sampling. Inspecting these trajectories indicates that the P4 pocket unbinds quickly and samples erratically at greater distances from the pocket at larger λ values, for ${{\varvec{q}}}_{3}^{W}$ and ${{\varvec{q}}}_{4}^{W}$ for WEHI-539 and ${{\varvec{q}}}_{1}^{A}$ for ABT-737, while the ligands remain bound to the P2 pocket. On the other hand, ${{\varvec{q}}}_{4}^{A}$ for ABT-737 unbinds more gradually, with both the P2 and P4 pockets dissociating at similar rates. Focusing on the configurations that are the most similar to the experimental ones, in the bound state (${{\varvec{q}}}_{3}^{W}$ for WEHI-539, Movie S1 and Fig. 3A, and ${{\varvec{q}}}_{1}^{A}$ for ABT-737, Movie S2 and Fig. 3B), the ligands in the P2 pocket are tightly packed and surrounded by phenylalanine residues (namely, Phe97, Phe105 and Phe146), forming π-π interactions with the ligands. At the lowest λ (see “Methods” section) values (deeper in the pocket than the global minimum), the ligand is pushed further into the pocket, especially for WEHI-539. As the ligands start to leave their pocket at the lower λ values, they first dissociate from the P4 pocket. WEHI-539 pulls free from the P2 pocket at about ${\uplambda }_{3}^{W}$=4.5 Å, while ABT-737 pulls free much later at around ${\uplambda }_{1}^{A}$=8 Å. At around ${\uplambda }_{1}^{A}$=9 Å the configurational sampling for ABT-737 becomes erratic, with just L2 contacting the protein and the remainder fluctuating in the bulk region. L2 of ABT-737 remains on the surface of the protein until about ${\uplambda }_{1}^{A}$=12.8 Å via hydrophobic interactions with e.g. Tyr101, Phe97 and Leu130, after which the ligand attains a wide assortment of random states, as it unbinds from the protein. For WEHI-539 on the other hand, after finally being able to leave the P2 pocket, the ensemble consists of multiple configurations, some similar to ${{\varvec{q}}}_{1}^{W}$, some that have L2 pack with the stem of Arg102, some that have L2 point towards P2. In each of these cases, the ligand interacts with Tyr101, but the residue has different rotameric states. At ${\uplambda }_{3}^{W}$=6.4 Å, L2 is positioned perpendicular to the pocket, with P4 in front of it on the outside of Bcl-xL. From this state, the ligand slowly dissociates, first L4, then slowly L2 until ${\uplambda }_{3}^{W}$=10.7 Å, at which point the configuration becomes erratic, with many different configurations being sampled and different parts of the ligand and the protein contacting as they slowly unbind.

## Discussion

We have executed two sets of independent dynamic docking and path sampling simulations on Bcl-xL with two different ligands, starting from the apo-state. The presence of the ligands shifts the balance of the conformational state of Bcl-xL in different manners, depending on the ligand. The largest difference is observed for WEHI-539, where the ligand forges a tunnel through P2 into the core of the protein, while ABT-737 binds on the surface. It is interesting to see the profound effects these ligands can have on the structural ensemble of the protein, as both simulations started from the same apo-state, but the ligand influenced the conformational ensemble considerably, indicative of a cryptic site. Large conformational changes, especially for WEHI-539, are required to enable the ligand to bind. However, the sporadic reweighted configurational ensemble with a low density of states along the binding pathway suggest that the binding event is sudden and thus follows a population shift pattern, where Bcl-xL first needs to attain a favorable state, before the ligand can bind. Computationally, dynamic docking of molecules that bind following a population shift paradigm can be difficult, as it’s a game of chance; when the molecules approach, the protein has to be in a reciprocal state to enable the ligand to bind, otherwise the binding will fail. Thus, not only the binding event must be enhanced, but also the conformational sampling of the individual molecules. McMD-based dynamic docking enables just that; it does not only enhance binding, but all dynamics within the system, including conformational sampling of the protein. Previously, Liu et al. showed that by using enhanced sampling simulations on the apo state of Bcl-xL, that the residues 98–120 showed high flexibility and their simulations sampled conformations similar to the apo state and multiple holo states^30^, also showing a population shift mechanism.

McMD is however not a magic bullet; the enhanced sampling also has some side effects in the case of binding mechanisms that follow the population shift model. Just as McMD enhances conformational and configurational sampling, it might also result in higher binding event failure rates because if the protein isn’t completely in an accommodating state, the ligand could quickly dissociate again, due the enhanced dynamics. This difficulty in combination with the large size of the ligands (leading to large conformational and configurational variability of the ligands) makes the binding of these ligands to Bcl-xL a challenging, yet interesting endeavor. Despite these odds, we were able to predict the bound configuration in agreement with the experimental structures for both ligands and achieved a very similar relative free energy for both complexes. By performing a long pre-run (750 ns per trajectory) a long production run (1 μs per trajectory) and a relatively large number of parallel simulations (N = 32), we were able to sample the conformational and configurational space sufficiently to predict intermediary and bound conformations.

**Figure 4:**
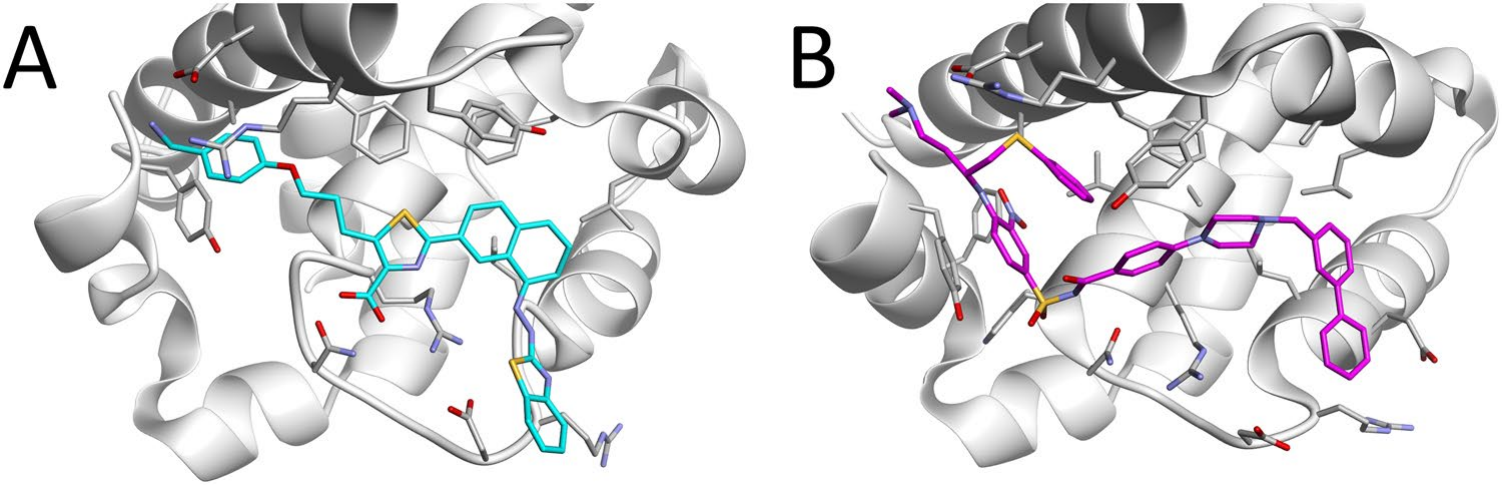
Comparison between ${r}_{1}^{W}$ (**A**) and Bcl-xL in complex with the ligand W1191542 (comp_id: X0J, PDB ID: 3INQ) (**B**). Also shown are the sidechains of Bcl-xL that are nearby the ligands. Both ligands attain a similar conformation, with L2 pointing outwards, positioning their rings between Glu129 and Asp133.

One of the major differences in binding of WEHI-539 and ABT-737, is that L2 of ABT-737 mainly binds within a shallow pocket on the surface of P2 of Bcl-xL, while L2 of WEHI-539 forges a tunnel within Bcl-xL, passing through P2, thus binding much deeper. This makes the binding process of WEHI-539 much more difficult than that of ABT-737, which can also be seen in our results (Tables 1 and 2). While our top rank (${{\varvec{r}}}_{1}^{A}$) for ABT-737 was close to the experimental structure, only ${{\varvec{r}}}_{3}^{W}$ for WEHI-539 has a structure similar to the experimental one. Here, for ${{\varvec{r}}}_{1}^{W}$, only L4 bound to P4 in a native state, while L2 was facing outwards, away from P2. Interestingly though, one of the binding attempts of the ligand during the McMD docking simulations actually had the ligand pass through ${{\varvec{r}}}_{1}^{W}$ to finally end up at ${{\varvec{r}}}_{3}^{W}$. In addition, our path sampling simulations also showed ${{\varvec{r}}}_{1}^{W}$ as one of the intermediary states. This suggests that ${{\varvec{r}}}_{1}^{W}$ is an actual intermediary state, before reaching ${{\varvec{r}}}_{3}^{W}$, where transition to ${{\varvec{r}}}_{1}^{W}$ requires considerably more effort and time. This state is actually very close to that of a different ligand (comp_id: X0J) that binds Bcl-xL, as shown by their crystal structure (PDB ID: 3INQ)^31^, where L2 isn’t inside the P2 sub-pocket, which has become very shallow in both cases (see Fig. 4), but pointing to the bulk, like ${{\varvec{r}}}_{1}^{W}$. This finding gives further credence that ${{\varvec{r}}}_{1}^{W}$ is one of the intermediary states in the binding process. The conformational change in the protein required to enable access to ${{\varvec{r}}}_{3}^{W}$ did not occur at a sufficiently high rate for ${{\varvec{r}}}_{3}^{W}$ to outrank ${{\varvec{r}}}_{1}^{W}$. NMR experiments have shown that the simple point mutation of F143W changes the redundancy of the P2 pocket to maintain the open state better^32^, which should lead to an increased association rate constant, k_on_, thus enabling the ligand to bind to Bcl-xL faster. Thus, taken together, such a mutation would most likely also decrease the population of configurations such as those found in 3INQ and ${{\varvec{r}}}_{1}^{W}$.

Our path sampling simulations found that ${{\varvec{q}}}_{3}^{W}$ of WEHI-539 and ${{\varvec{q}}}_{1}^{A}$ of ABT-737 have a slightly stronger affinity ${\mathrm{\Delta G}}_{\mathrm{b}}^{0}$ (−17.95 kcal/mol and −15.45 kcal/mol, for ${{\varvec{q}}}_{3}^{W}$ and ${{\varvec{q}}}_{1}^{A}$, respectively) compared to the experimental affinity^14^ (−12.66 kcal/mol and −12.90 kcal/mol for WEHI-539 and ABT-737, respectively), in particular WEHI-539. The experimental data suggested that WEHI-539 has both faster association and dissociation rates compared to ABT-737^14^, however given the more complex structure of the WEHI-539/Bcl-xL complex^14^, this seems rather peculiar. Our dynamic docking simulations however suggested that a different configuration, ${{\varvec{r}}}_{1}^{W}$, was the most stable one, while on the other hand, our path sampling simulations showed that the affinity ${\mathrm{\Delta G}}_{\mathrm{b}}^{0}$ of ${{\varvec{q}}}_{1}^{W}$ is lower than that of ${{\varvec{q}}}_{3}^{W}$, suggesting that although the affinity of ${{\varvec{r}}}_{3}^{W}$ is better, the association rate is much slower. On the other hand, for ABT-737, configuration ${{\varvec{q}}}_{2}^{A}$ showed a higher ${\mathrm{\Delta G}}_{\mathrm{b}}^{0}$ of −16.12 kcal/mol compared to ${{\varvec{q}}}_{1}^{A}$, which is also peculiar, as the canonical simulations at 400 K showed that it was considerably less stable than ${{\varvec{q}}}_{1}^{A}$. Potentially, the hydrophobic interactions that the ligand in ${{\varvec{q}}}_{2}^{A}$ makes in this deeply embedded configuration, which has a similar relative accessible surface area (RASA) to ${{\varvec{q}}}_{3}^{W}$, are quite stable at 300 K even as the ligand unbinds, but destabilize at higher temperatures due to the increased entropy.

In summary, we have used McMD-based dynamic docking simulations to compare the binding mechanism between two medium-sized inhibitors that bind to the cryptic site of Bcl-xL. We were able to predict binding configurations similar to the experimental ones, despite the large size of the ligands compounded with the increased difficulty due to the cryptic nature of the pocket. We also predicted the binding free energy of the two inhibitors, with the affinity of ABT-737 matching that of the experimental value and that of WEHI-539 being slightly higher. Furthermore, our simulations show that the conformational preference of Bcl-xL is modulated, depending on the ligand, as we started with the same apo state for both simulations, and were able to obtain different conformations for the protein in the bound states. For both compounds, we observed a population shift upon binding, where WEHI-539 required additional time to bind to switch from ${{\varvec{q}}}_{1}^{W}$ to ${{\varvec{q}}}_{3}^{W}$ (binding of P2, after P4 had already bound), due to a narrow transition pathway between the intermediary and native bound state. We have shown that McMD-based dynamic docking can be a useful tool to study the binding mechanism, even in the case of a cryptic pocket, where ligand binding can shift the major conformational population of the protein.

## Methods

### Computational systems

The apo structure of Bcl-xL in its monomeric state with PDB ID 1R2D (resolution 1.95 Å) was obtained from Protein Data Bank Japan (PDBj)^33–35^. The missing Ser28-Ile81 loop region was replaced by an 8-mer Gly linker using MODELLER^36^. The ligands WEHI-539 (comp_id X0B)^14^ and ABT-737 (comp_id N3C)^13^ used for the docking, and the corresponding holo structures, which were only used to confirm the docking results, PDB IDs 3ZLR and 2YXJ, respectively, were also obtained from PDBj. We used Gromacs 2020.1^37^ to prepare and perform the simulations, which we modified to perform the dynamic docking and path sampling simulations^16,18–21^. The apo structure was first aligned using its principal axis of inertia with the x-axis of a triclinic box that was placed around the system, with the minimum distance from the protein to the edge of the box set to be at least 12 Å (giving a box of size 68.6 Å × 62.7 Å × 61.7 Å). The box was solvated, with Na^+^ and Cl^−^ added to neutralize the system and bring the concentration to physiological levels (0.1 M). The Amber99SB-ILDN force field^38^, GAFF2^39^, monovalent ion parameters^40^ and TIP3P^41^ were used to parameterize the protein, the ligands, ions and water molecules, respectively. For each of the ligand, Gaussian^42^ at the HF/6-31G* level was used to optimize the geometries and calculate the electron density, followed by RESP^43,44^ to finally obtain the atomic partial charges by fitting the density. The ligands were placed inside the box, at a distance of about 15 Å from the protein, before the solvent was added. The final system using the ligand WEHI-539 consisted of 2307 protein atoms, 70 ligand atoms, 7840 water molecules, 21 Na ions and 16 Cl ions. The final system using the ligand ABT-737 consisted of 2307 protein atoms, 101 ligand atoms, 7833 water molecules, 21 Na ions and 16 Cl ions.

NVT simulations were performed at 300 K using the Bussi thermostat^45^, while the NPT simulations additionally used the Parrinello-Rahman barostat^46^ under 1 bar at 300 K. The long-range electrostatics were calculated using the zero-dipole summation method, which is a cutoff-based approach utilizing a well-defined pairwise function^18,47,48^, with the damping factor α set to 0 Å^−1^ and the atom-based cutoff length set to 12 Å. A time-step of 2 fs was used, with LINCS^49^ to constrain the bond lengths and SETTLE^50^ to constrain the water geometries. Energy minimizations, followed by 100 ps NVT and NPT simulations with position restraints on the heavy solute atoms were used to prepare the system.

### Dynamic docking

To prevent unfolding at high temperatures during the McMD simulations and the initial high temperature dissociation simulation, we employed distance restraints. Although in our most recent paper^21^ we employed flat-bottomed position restraints on the protein’s Cα atoms that form the secondary structure, since we wanted to ensure sufficient flexibly for the α2-α4 region, which differs considerably between all the structures, we went with a different approach. Here, given the apo structure, we used distance restraints between the backbone oxygen and nitrogen atoms for the residues that form hydrogen bonds that stabilize the secondary structure (in the case of Bcl-xL, only α-helices), with a flat-bottom region spanning between 0 and 4.5 Å, after which a 10 kcal/mol/Å^2^ force constant is used to restrain the hydrogen bond. As these restraints only prevent the secondary structure from breaking at high temperature, but not the tertiary structure, we’ve also added restraints between 9 Cα atom pairs (Arg91-Asp11, Leu13-Gly147, Leu13-Ala167, Glu133-His177, Tyr195-Ala89, Val141-Ala93, Pro116-Val161, Ser2-Asn175, Val135-Trp181). These were restrained using a flat-bottom potential at ± 2 Å from their measured distance in the apo structure using a 10 kcal/mol/Å^2^ force constant. In addition, the translation and rotation of the center of mass (COM) of the protein was also restrained^19^ to keep the protein centered inside the box during the simulations.

Our docking protocol is similar to that used in our recently published paper of exhaustive sampling of the configurational space of Hsp90^21^, where the ligand can access the whole domain. Both the WEHI-539 and ABT-737 ligand systems start from the same initial structure, i.e. the apo conformation of Bcl-xL, using the same restraints on the protein. We used the McMD algorithm described in Section S1 on both of the systems, with 32 parallel trajectories initialized with different random seeds for the initial velocity from the initial structure constructed in section 1. After the initialization, first a 1 ns simulation at *T*_high_ (= 700 K) was performed for each parallel trajectory to randomize the ensemble with the above described weak distance restraints in place. From here, the initial bias was estimated using Eq. S3 and subsequent iterations of increasing simulation lengths were executed, updating the bias using Eq. S4 between iterations, until a sufficiently flat potential energy distribution had been obtained corresponding to a wide *T*_mc_ range of *T*_low_ − *T*_high_, where *T*_low_ = 280 K. In total, the pre-run lasted for 24 µs (750 ns per trajectory) per system. Finally, a 32 µs (1 µs per trajectory) production run was executed to sample the structures including bound and unbound states, which were saved at 5 ps intervals, producing 6.4 × 10^6^ structures for each system. Both systems were simulated for exactly the same simulation length.

### Dynamic docking analysis

Since the configurational space was exhaustively sampled, we used the analysis techniques introduced in our most recent work on each of the systems^21^. First, we performed PCA on a distance array derived from the structures. The array consists of protein–ligand pairs and ligand-ligand pairs. For the protein atoms, its Cα atoms were taken and paired with the ligand atoms indicated by a “*” symbol in Fig. S9 (inter-molecular) and Fig. S10 for the ligands WEHI-539 and ABT-737, respectively. For the protein, we excluded the N-terminal region (37 residues), as they differ significantly between all the structures and our structure includes a modelled Gly-linker, which combined, makes comparison to the experimental structures more difficult. Furthermore, the atoms indicated by characters in each of the figures form the ligand-ligand pairs (intra-molecular). The distance based approach does not require prior superposition of the structures unlike the quasi-harmonic approach^24,51^, while taking periodic boundary conditions into account and being a more sensitive approach to detecting intermolecular contacts along the entire surface between the interacting molecules. The structures are then projected onto the first two principal components, and the probability of each bin *i* on the landscape is calculated as ${P}_{i}={\sum }_{j}{P}_{c}\left({E}_{j}, 300 K\right)$ using each structure *j* within bin *i*. The free energy is finally calculated as ${PMF}_{i}=-RTln {P}_{i}$ for each bin, giving the 2D FEL after normalizing its minimum to zero.

After the PCA, we performed K-means clustering on the data, using *k*′ = 1000 clusters and a number of PC coordinates, so that the sum of the contribution to their variance exceeds 90%, corresponding to PC1-PC8 and PC1-PC10 for the WEHI-539 and ABT-737 systems, respectively (see Fig. 2A,B). For each cluster, one representative structure was then selected and were then ranked based on the relative free energy at 300 K of the clusters, CFE, which was calculated as ${PMF}_{{k}^{^{\prime}}}=-RTln {P}_{{k}^{^{\prime}}}$, where ${P}_{{k}^{^{\prime}}}={\sum }_{j}{P}_{c}\left({E}_{j}, 300 \mathrm{K}\right)$ using each structure *j* corresponding to cluster *k’*. Then, using the representative structures, direct analysis on the structural properties is performed using R-value analysis^19,21^. Starting from the most stable cluster *k*′ = 1 in order of their free energy contribution, similar representative structures in terms of their intermolecular contacts calculated via the R-value (R > 0.7) were grouped together. After calculating the free energy contribution of the grouped clusters, we used a CFE cutoff value of 1.5 kcal/mol to distinguish between potentially interesting structures and less stable structures. This gave us *k* number of clusters and their corresponding representative structures **r**_k_, for each system.

Representative complex structures **r**_k_ obtained from the multicanonical ensemble were further refined using canonical (NVT) MD simulations at 300 K. For each representative structure, ten 100 ns MD simulations at 300 K were performed (with different random seeds for the initial velocity). Then, refined complex structures **q**_k_ were picked by taking the nearest-to-average structure from the final 40 ns of the canonical MD simulations. In addition, 400 K canonical MD simulations were performed to compare the relative stabilities of the binding configurations, like we have done before^19–21^.

### Binding pathway analysis

We used our previously developed pathing method^19–21^ to construct a binding pathway starting from each refined bound configurations **q**_k_. As we did not use a cylinder to restrain the sampling region of the ligand, we did not have a reaction coordinate to use for the pathing method. Previously, we developed a naïve method to estimate the optimal unbinding direction of a ligand, given its size and the shape of the pocket^18^. Here, we have applied this algorithm on each configuration **q**_k_, producing a different reaction coordinate λ (estimated association/dissociation direction) for each configuration. Then, using the configuration and the corresponding reaction coordinate, we used our pathing algorithm to construct the binding/unbinding pathway. In short (see Section S2 for a longer description), the reaction coordinate is split into pre-defined windows. For the window corresponding to the bound state, similar structures to the bound configuration are taken from the multicanonical ensemble based on our R-value metric^19^, clustered using K-means clustering (with *k* = 3), and one representative structure is picked for each cluster (while considering the multicanonical weight of each of the structures part of the cluster). For the first window, the picked structure that is the most similar (i.e. has the highest R-value to **q**_k_) is replaced with **q**_k_ in the set of representative structures for a window. The R-value simply measures the fraction of representative contacts between the protein and ligand with respect to a reference structure, based on the Q-value^28,29^, the fraction of native contacts. In the neighboring windows, the same picking algorithm is used, but the structures in the window are compared with the previously picked structures. Thus, for each subsequent window, the comparison structures become those picked for the preceding window. Once the ligand approaches the unbound state, instead of using the R-value, the RMSD_L_ (RMSD that ignores the translation along the reaction coordinate) is used instead, as there are not a sufficient number of intermolecular contacts remaining. Thus, starting from **q**_k_, the above algorithm picks representative structures at specific intervals from the multicanonical ensemble that provides a relatively smooth transition in intermolecular contacts between the ligand and the protein, while still allowing some variability (by picking three representative structures for each window)^19^.

To analyze the binding pathway in greater detail, we performed path sampling simulations based on the Umbrella Sampling (US) method. Using the representative structures obtained via our pathing method, we first performed a short 100 ps canonical MD simulation with position restraints on all heavy solute atoms at 400 K, followed by 2000 ps of US at 400 K, where during the final 200 ps, the temperature was slowly annealed to 300 K. Our previous work showed that short temperatures at 400 K enables the system to more quickly escape local minima, enabling it to attain a more stable state^19^. Finally, for the production run, 250 ns of US simulations at 300 K for each window was performed. During these simulations, the ligand’s COM was restrained perpendicularly inside a cylinder (with a radius of 8 Å, and a force constant of 0.1 kcal/mol/Å^2^) and to the center of the window that it was picked from (with an adaptive force constant, see Table S12) along the reaction coordinate λ defined by our naïve method. The protein’s COM was restrained in the same manner as for the dynamic docking simulations, but no distance restraints, like those used during the McMD simulations, were used. During the production run, a snapshot was saved at every 10 ps, with the COM of the ligand saved at every 1 ps. To calculate the affinity, the Weighted Histogram Method (WHAM) was used^52^. WHAM was performed to a tolerance of 1e^−8^ using various amounts of the trajectory data with 1000 bootstraps. For ${{\varvec{r}}}_{1}^{W}$, the final three windows were omitted, as they produced structures that would have the ligand and protein approaching each other, due to periodic boundary conditions.

## Supplementary Information

Supplementary Information.

## Data Availability

The representative structures and interactive versions of Figs. 1, 2C,D, 3, 4, S5 and S6 have been submitted to the Biological Structure Model Archive (BSM-Arc)^53^, under BSM-00021 (https://bsma.pdbj.org/entry/21).

## Code availability

The source code for executing McMD dynamic docking simulations, including a modified version of Gromacs and the analysis scripts, are available at https://gitlab.com/gjbekker/gromacs.

## References

[1] Chao DT, Korsmeyer SJ (1998). BCL-2 FAMILY: regulators of cell death. Annu. Rev. Immunol..

[2] Czabotar PE, Lessene G, Strasser A, Adams JM (2014). Control of apoptosis by the BCL-2 protein family: implications for physiology and therapy. Nat. Rev. Mol. Cell Biol..

[3] Boise LH (1993). bcl-x, a bcl-2-related gene that functions as a dominant regulator of apoptotic cell death. Cell.

[4] Sattler M (1997). Structure of Bcl-xL-Bak peptide complex: recognition between regulators of apoptosis. Science.

[5] Petros AM (2000). Rationale for Bcl-XL/Bad peptide complex formation from structure, mutagenesis, and biophysical studies. Protein Sci..

[6] Liu X, Dai S, Zhu Y, Marrack P, Kappler JW (2003). The structure of a Bcl-xL/Bim fragment complex. Immunity.

[7] Hanahan D, Weinberg RA (2011). Hallmarks of cancer: the next generation. Cell.

[8] Cimermancic P (2016). CryptoSite: expanding the druggable proteome by characterization and prediction of cryptic binding sites. J. Mol. Biol..

[9] Iida S, Nakamura HK, Mashimo T, Fukunishi Y (2020). Structural fluctuations of aromatic residues in an apo-form reveal cryptic binding sites: implications for fragment-based drug design. J. Phys. Chem. B.

[10] Lee EF, Fairlie WD (2019). The structural biology of Bcl-xL. Int. J. Mol. Sci..

[11] Muchmore SW (1996). X-ray and NMR structure of human Bcl-xL, an inhibitor of programmed cell death. Nature.

[12] Oltersdorf T (2005). An inhibitor of Bcl-2 family proteins induces regression of solid tumours. Nature.

[13] Lee EF (2007). Crystal structure of ABT-737 complexed with Bcl-xL: implications for selectivity of antagonists of the Bcl-2 family. Cell Death Differ..

[14] Lessene G (2013). Structure-guided design of a selective BCL-XL inhibitor. Nat. Chem. Biol..

[15] Gioia D, Bertazzo M, Recanatini M, Masetti M, Cavalli A (2017). Dynamic docking: a paradigm shift in computational drug discovery. Molecules.

[16] Bekker, G.-J. & Kamiya, N. Dynamic docking using multicanonical molecular dynamics: simulating complex formation at the atomistic level. In *Protein-Ligand Interactions and Drug Design* (ed. Ballante, F.) (Springer, 2021). 10.1007/978-1-0716-1209-5_11. 10.1007/978-1-0716-1209-5_11 33759128

[17] Kamiya N, Yonezawa Y, Nakamura H, Higo J (2008). Protein-inhibitor flexible docking by a multicanonical sampling: native complex structure with the lowest free energy and a free-energy barrier distinguishing the native complex from the others. Proteins.

[18] Bekker G-J (2017). Accurate prediction of complex structure and affinity for a flexible protein receptor and its inhibitor. J. Chem. Theory Comput..

[19] Bekker G-J, Araki M, Oshima K, Okuno Y, Kamiya N (2019). Dynamic docking of a medium-sized molecule to its receptor by multicanonical MD simulations. J. Phys. Chem. B.

[20] Bekker G-J, Fukuda I, Higo J, Kamiya N (2020). Mutual population-shift driven antibody-peptide binding elucidated by molecular dynamics simulations. Sci. Rep..

[21] Bekker G-J, Araki M, Oshima K, Okuno Y, Kamiya N (2020). Exhaustive search of the configurational space of heat-shock protein 90 with its inhibitor by multicanonical molecular dynamics based dynamic docking. J. Comput. Chem..

[22] Nakajima N, Higo J, Kidera A, Nakamura H (1997). Flexible docking of a ligand peptide to a receptor protein by multicanonical molecular dynamics simulation. Chem. Phys. Lett..

[23] Nakajima N, Nakamura H, Kidera A (1997). Multicanonical ensemble generated by molecular dynamics simulation for enhanced conformational sampling of peptides. J. Phys. Chem. B.

[24] Kamiya N, Higo J, Nakamura H (2002). Conformational transition states of a β-hairpin peptide between the ordered and disordered conformations in explicit water. Protein Sci..

[25] Kamiya N, Mitomo D, Shea JE, Higo J (2007). Folding of the 25 residue Aβ(12–36) peptide in TFE/water: temperature-dependent transition from a funneled free-energy landscape to a rugged one. J. Phys. Chem. B.

[26] Nishigami H, Kamiya N, Nakamura H (2016). Revisiting antibody modeling assessment for CDR-H3 loop. Protein Eng. Des. Sel..

[27] Kitao A, Hirata F, Go N (1991). The effects of solvent on the conformation and the collective motions of protein—normal mode analysis and molecular-dynamics simulations of melittin in water and in vacuum. Chem. Phys..

[28] Best RB, Hummer G, Eaton WA (2013). Native contacts determine protein folding mechanisms in atomistic simulations. Proc. Natl. Acad. Sci. USA.

[29] Bekker G-J, Ma B, Kamiya N (2019). Thermal stability of single-domain antibodies estimated by molecular dynamics simulations. Protein Sci..

[30] Liu X, Jia Z, Chen J (2017). Enhanced sampling of intrinsic structural heterogeneity of the BH3-only protein binding interface of Bcl-xL. J. Phys. Chem. B.

[31] Lee EF (2009). Conformational changes in Bcl-2 Pro-survival proteins determine their capacity to bind ligands. J. Biol. Chem..

[32] Mizukoshi Y (2020). Targeting the cryptic sites: NMR-based strategy to improve protein druggability by controlling the conformational equilibrium. Sci. Adv..

[33] Kinjo AR (2017). Protein Data Bank Japan (PDBj): updated user interfaces, resource description framework, analysis tools for large structures. Nucleic Acids Res..

[34] Kinjo AR (2018). New tools and functions in data-out activities at Protein Data Bank Japan (PDBj). Protein Sci..

[35] Burley SK (2019). Protein Data Bank: the single global archive for 3D macromolecular structure data. Nucleic Acids Res..

[36] Webb B, Sali A (2016). Comparative protein structure modeling using MODELLER. Curr. Protoc. Bioinform..

[37] Kutzner C (2019). More bang for your buck: improved use of GPU nodes for GROMACS 2018. J. Comput. Chem..

[38] Lindorff-Larsen K (2010). Improved side-chain torsion potentials for the amber ff99SB protein force field. Proteins.

[39] Wang J, Wolf RM, Caldwell JW, Kollman PA, Case DA (2004). Development and testing of a general amber force field. J. Comput. Chem..

[40] Joung IS, Cheatham TE (2008). Determination of alkali and halide monovalent ion parameters for use in explicitly solvated biomolecular simulations. J. Phys. Chem. B.

[41] Jorgensen WL, Chandrasekhar J, Madura JD, Impey RW, Klein ML (1983). Comparison of simple potential functions for simulating liquid water. J. Chem. Phys..

[42] Frisch, M. J. *et al.* Gaussian 09, revision D.01.

[43] Bayly CI, Cieplak P, Cornell WD, Kollman PA (1993). A well-behaved electrostatic potential based method using charge restraints for deriving atomic charges: the RESP model. J. Phys. Chem..

[44] Cornell WD, Cieplak P, Bayly CI, Kollman PA (1993). Application of RESP charges to calculate conformational energies, hydrogen bond energies, and free energies of solvation. J. Am. Chem. Soc..

[45] Bussi G, Donadio D, Parrinello M (2007). Canonical sampling through velocity rescaling. J. Chem. Phys..

[46] Parrinello M, Rahman A (1981). Polymorphic transitions in single-crystals—a new molecular-dynamics method. J. Appl. Phys..

[47] Fukuda I, Yonezawa Y, Nakamura H (2011). Molecular dynamics scheme for precise estimation of electrostatic interaction via zero-dipole summation principle. J. Chem. Phys..

[48] Kamiya N, Fukuda I, Nakamura H (2013). Application of zero-dipole summation method to molecular dynamics simulations of a membrane protein system. Chem. Phys. Lett..

[49] Hess B (2008). P-LINCS: a parallel linear constraint solver for molecular simulation. J. Chem. Theory Comput..

[50] Miyamoto S, Kollman PA (1992). Settle—an analytical version of the shake and rattle algorithm for rigid water models. J. Comput. Chem..

[51] Numoto N (2018). Structural dynamics of the PET-degrading cutinase-like enzyme from *Saccharomonospora viridis* AHK190 in substrate-bound states elucidates the Ca^2+^ -driven catalytic cycle. Biochemistry.

[52] Grossfield, A. WHAM: an implementation of the weighted histogram analysis method.

[53] Bekker G-J, Kawabata T, Kurisu G (2020). The Biological Structure Model Archive (BSM-Arc): an archive for in silico models and simulations. Biophys. Rev..

[54] Bekker G-J, Nakamura H, Kinjo AR (2016). Molmil: a molecular viewer for the PDB and beyond. J. Cheminform..

