# Supplementary material for: Cryptic-site binding mechanism of medium-sized Bcl-xL inhibiting compounds elucidated by McMD-based dynamic docking simulations
Source: Sci Rep. 2021 Mar 3;11:5046. doi: 10.1038/s41598-021-84488-z (PMC7930018; doi:10.1038/s41598-021-84488-z)
Supplement: Supplementary file 1 — Supplementary Information. [file 41598_2021_84488_MOESM1_ESM.pdf]

Cryptic-site binding mechanism of medium-sized Bcl-xL inhibiting compounds elucidated by McMD-based dynamic docking simulations

Gert-Jan Bekker[1,*], **Ikuo Fukuda**[2], **Junichi Higo**[2], **Yoshifumi Fukunishi**[3] and **Narutoshi Kamiya**[2,*]

[1] Institute for Protein Research, Osaka University, 3-2 Yamadaoka, Suita, Osaka 565-0871, Japan.

[2] Graduate School of Simulation Studies, University of Hyogo, 7-1-28 Minatojima Minami-machi, Chuo-ku, Kobe, Hyogo 650-0047, Japan.

[3]Cellular and Molecular Biotechnology Research Institute, National Institute of Advanced Industrial Science and Technology (AIST), 2-3-26, Aomi, Koto-ku, Tokyo 135-0064, Japan.

Table of contents

**Section S1**	McMD algorithm	3
**Section S2**	Binding mechanism analysis	4
**Table S1**	Relative stability of the binding configurations for the ligand WEHI-539	7
**Table S2**	Relative stability of the binding configurations for the ligand ABT-737	8
**Table S3**	Picking statistics ($r_1^W, r_2^W, r_3^W, r_4^W, r_1^A, r_2^A, r_3^A, r_4^A$).	9
**Table S4**	Free energy obtained for ligand WEHI-539 in configuration q_1	18
**Table S5**	Free energy obtained for ligand WEHI-539 in configuration q_2	19
**Table S6**	Free energy obtained for ligand WEHI-539 in configuration q_3	20
**Table S7**	Free energy obtained for ligand WEHI-539 in configuration q_4	21
**Table S8**	Free energy obtained for ligand ABT-737 in configuration q_1	22
**Table S9**	Free energy obtained for ligand ABT-737 in configuration q_2	23
**Table S10**	Free energy obtained for ligand ABT-737 in configuration q_3	24
**Table S11**	Free energy obtained for ligand ABT-737 in configuration q_4	25
**Table S12**	Window characteristics	26
**Figure S1**	WEHI-539 McMD potential energy distribution	27
**Figure S2**	ABT-737 McMD potential energy distribution	28
**Figure S3**	Location of picked representative configurations r_k^W on the FEL	29
**Figure S4**	Location of picked representative configurations r_k^A on the FEL	30
**Figure S5**	3D structure of picked representative configurations r_k^W	31
**Figure S6**	3D structure of picked representative configurations r_k^A	32
**Figure S7**	PMF profile for each picked representative configurations q_k^W	33
**Figure S8**	PMF profile for each picked representative configurations q_k^A	34
**Figure S9**	2D structure of the ligand WEHI-539	35
**Figure S10**	2D structure of the ligand ABT-737	35
**Figure S11**	WEHI-539 McMD potential energy distribution	36
**Movie S1**	https://www.youtube.com/watch?v=ukkI_g8XqpM	
**Movie S2**	https://www.youtube.com/watch?v=a7tds4dFHMk	

Section S1: McMD algorithm

We used our developed McMD-based dynamic docking method that has been thoroughly described in various previous papers,[1–9] but here we will shortly review the algorithm. The probability distribution of the potential energy of the multicanonical ensemble is defined by the following equation:

$$P_{mc}(E,T_0) = \frac{1}{Z_{mc}} n(E) e^{-W(E)} = constant \quad \text{(S1)}$$

where E is the potential energy, T_0 the simulation temperature, $n(E)$ the density of states and Z_{mc} the partition function:

$$Z_{mc} = \int n(E) e^{-W(E)} dE \quad \text{(S2)}$$

$W(E)$ is a weighting function to modulate the probability distribution P_{mc} in order for it to become constant and enables the system to take a random walk along the target energy range, and is defined as follows:

$$W(E) = \ln n(E) = \frac{E}{RT_0} + \ln P_c(E,T_0) \quad \text{(S3)}$$

where R is the gas constant and P_c the canonical energy distribution at T_0. During the McMD simulations, this weighting function is used to scale the forces by a factor of $\nabla W(E)$, where the multicanonical temperature T_{mc}, which corresponds to $T_0/\nabla W(E)$, is restricted to a specific target range between T_{low} to T_{high}, which we generally set to 280 K and 700 K, respectively. Multiple iterations of sampling are required to estimate the correct bias that enables a random walk along a wide energy range, where the weighting function is updated between iterations using:

$$W^{i+1}(E) = W^i(E) + \ln P_{mc}^i(E,T_0) \quad \text{(S4)}$$

After obtaining a flat potential energy distribution, a production run is executed to sample phase space. Due to the bias applied during the McMD simulations, the resulting multicanonical ensemble must be reweighted to obtain the canonical distribution at room temperature. A multicanonical distribution can be reweighted to a canonical distribution at any given temperature T within the flat energy range using the following equation:

$$P_c(E,T) = \frac{1}{Z_c} n(E) e^{-\frac{E}{RT}} = \frac{Z_{mc}}{Z_c} P_{mc}(E) e^{W(E)-\frac{E}{RT}} \quad \text{(S5)}$$

Section S2: Binding mechanism analysis

Using the methodology we previously described,[10,11] we picked structures along the estimated binding/unbinding direction. There we used a cylinder to limit the sampling during the McMD-based dynamic docking, and coincidentally used that direction as the guiding direction for the estimation for the smooth, connected path. However, since we did not use a cylinder here, we could not use its direction for as the guide for our picking algorithm. We previously introduced a naïve algorithm for estimating the binding direction given the center of the pocket.[8] Here, instead of using the algorithm on our initial structure with an approximate location of the pocket, we can use any of the structures $\mathbf{q}_k$ as the initial structure, with the pocket defined as the center of mass of the ligand in the bound state in the predicted structure to generate the dissociation axis $\vec{\lambda}_k$.

We previously described our structure picking method that can pick structures from an McMD trajectory to generate a smooth path in our recent docking between β-secretase and its inhibitor[10]. For clarity we will shortly review it here. First, we defined a number of windows (and their sizes) from which to pick representative structures from, where we picked 3 unique structures per window to increase the statistics of the US simulations. For the picking process, each window is defined by the parameters λ'_l, λ'_c, λ'_u along $\vec{\lambda}$ as described in Table S12. For each window, we first obtain a set of eligible structures by filtering the entire McMD ensemble based on the λ' range λ'_l - λ'_u. Next, we filter them based on the structural similarity with respect to the picked structures from the preceding window via a nearest neighbor like approach, obtaining a filtered ensemble. Finally, using this subset, we performed K-means clustering (with k=3) where we take a representative structure from each cluster. This leads to an optimally structurally connected ensemble of structures along λ' from the predicted bound configuration to the unbound state, given the McMD ensemble and a starting configuration. Here, we have performed this procedure on all the structures $\mathbf{q}_k$ obtained for both systems that have a cluster free energy value of less or equal to 0.5 kcal/mol, to study their binding/unbinding pathways.

To sample the interactions between the ligands and Bcl-xL in greater detail, we performed path sampling simulations starting from the picked configurations. First, an energy minimization stage was performed until the maximum force was less than 100 kJ/mol/nm^2 to stabilize the structures (as the structures were picked from the multicanonical ensemble, their potential energy varies greatly). Next, a 100 ps canonical MD simulation was performed, where the velocities were initialized according to a Maxwell distribution corresponding to 300 K. Finally, a 200 ns path sampling simulation at 300 K was performed for each of the picked structures, with the center of mass (COM) of the ligand restrained perpendicularly within a cylinder with a radius of 8 Å, whose axis $\vec{\lambda}_k$ was defined by the naïve method as described above, and restrained to the center of the corresponding window (see Table S12 for the window parameters). Like our previous method[10,11] we employed a short high-temperature simulation in order to more quickly leave local minima and attain more stable structures. The translation and rotation of the COM of Bcl-xL were restrained as described above, without any position or distance restraints. Finally, the PMF along $\vec{\lambda}_k$ was calculated using the Weighted Histogram Analysis Method (WHAM)[12,13], which was performed using a bin size $\Delta\lambda$ of 0.05 Å, a tolerance of 1e^{-8} and with 1000 bootstraps to estimate the error, where the binding free energy ΔG was then calculated as follows:

$$\Delta G = -\big(G(\lambda_\infty) - G(\lambda_0)\big) \tag{S6}$$

where $G(\lambda_\infty)$ corresponds to the estimate in the unbound state and $G(\lambda_0)$ in the bound state. The wide sampling of the US simulations enables us to calculate the sampled volume directly from these simulations to apply the correction term $e^{-\beta G(\lambda,v,\zeta)}$ from the probability distribution of the ensemble sampled during the US simulations, weighted by PMF obtained from WHAM, i.e., the distribution along (v,ζ) was weighted by $G(\lambda)$. Here, v and ζ correspond to the axes perpendicular to $\vec{\lambda}$ and the 3D grid consists of cubic cells of size $\Delta\lambda = 0.05$ Å. Finally, the standard binding free energy ΔG_b^0 was estimated using the following equation:

$$\Delta G_b^0 = \Delta G - k_B T \times \ln\Big(\frac{1}{V_0}\sum_{site} e^{-\beta G(\lambda,v,\zeta)}\Delta\lambda\Delta v\Delta\zeta\Big) \tag{S7}$$

where ΔG corresponds to the binding free energy calculated in Eq. S6, V_0 to the standard concentration at 1 mol/L (1661 Å^3), $e^{-\beta G(\lambda,v,\zeta)}$ to the probability of sampling inside the 3D grid cell (λ, v, ζ) and $\Delta\lambda\Delta v\Delta\zeta$ to the volume of the grid cell.

REFERENCES

(1) Nakajima, N.; Nakamura, H.; Kidera, A. Multicanonical Ensemble Generated by Molecular Dynamics Simulation for Enhanced Conformational Sampling of Peptides. *J. Phys. Chem. B* **1997**, *101* (5), 817–824.

(2) Nakajima, N.; Higo, J.; Kidera, A.; Nakamura, H. Free Energy Landscapes of Peptides by Enhanced Conformational Sampling. *J. Mol. Biol.* **2000**, *296* (1), 197–216.

(3) Higo, J.; Ito, N.; Kuroda, M.; Ono, S.; Nakajima, N.; Nakamura, H. Energy Landscape of a Peptide Consisting of α-Helix, 310-Helix, β-Turn, β-Hairpin, and Other Disordered Conformations. *Protein Sci.* **2001**, *10* (6), 1160–1171.

(4) Kamiya, N.; Higo, J.; Nakamura, H. Conformational Transition States of a β-Hairpin Peptide Between the Ordered and Disordered Conformations in Explicit Water. *Protein Sci.* **2002**, *11* (10), 2297–2307.

(5) Ikeda, K.; Higo, J. Free-Energy Landscape of a Chameleon Sequence in Explicit Water and Its Inherent α/β Bifacial Property. *Protein Sci.* **2003**, *12* (11), 2542–2548.

(6) Kamiya, N.; Yonezawa, Y.; Nakamura, H.; Higo, J. Protein-Inhibitor Flexible Docking by a Multicanonical Sampling: Native Complex Structure with the Lowest Free Energy and a Free-Energy Barrier Distinguishing the Native Complex from the Others. *Proteins* **2008**, *70* (1), 41–53.

(7) Ikebe, J.; Umezawa, K.; Kamiya, N.; Sugihara, T.; Yonezawa, Y.; Takano, Y.; Nakamura, H.; Higo, J. Theory for Trivial Trajectory Parallelization of Multicanonical Molecular Dynamics and Application to a Polypeptide in Water. *J. Comput. Chem.* **2011**, *32* (7), 1286–1297.

(8) Bekker, G.-J.; Kamiya, N.; Araki, M.; Fukuda, I.; Okuno, Y.; Nakamura, H. Accurate Prediction of Complex Structure and Affinity for a Flexible Protein Receptor and Its Inhibitor. *J. Chem. Theory Comput.* **2017**, *13* (6), 2389–2399.

(9) Bekker, G.-J.; Kamiya, N. Dynamic Docking Using Multicanonical Molecular Dynamics: Simulating Complex Formation at the Atomistic Level. In *Protein-Ligand Interactions and Drug Design*; Ballante, F., Ed.; Springer, 2021.

(10) Bekker, G.-J.; Araki, M.; Oshima, K.; Okuno, Y.; Kamiya, N. Dynamic Docking of a Medium-Sized Molecule to Its Receptor by Multicanonical MD Simulations. *J. Phys. Chem. B* **2019**, *123* (11), 2479–2490.

(11) Bekker, G.-J.; Fukuda, I.; Higo, J.; Kamiya, N. Mutual Population-Shift Driven Antibody-Peptide Binding Elucidated by Molecular Dynamics Simulations. *Sci. Rep.* **2020**, *10* (1), 1406.

(12) Kumar, S.; Bouzida, D.; Swendsen, R. H.; Kollman, P. A.; Rosenberg, J. M. The Weighted Histogram Analysis Method for Free-Energy Calculations on Biomolecules. I. The Method. *J. Comput. Chem.* **1992**, *13* (8), 1011–1021.

(13) Grossfield, A. WHAM: An Implementation of the Weighted Histogram Analysis Method. 2.0.9.

Table S1. Relative stability of the binding configurations for the ligand WEHI-539.

	R-value [a] 300 K	R-value [a] 400 K
$\mathbf{r}_1$	0.966 (0.038)	0.769 (0.168)
$\mathbf{r}_2$	0.906 (0.092)	0.717 (0.159)
$\mathbf{r}_3$	0.976 (0.022)	0.829 (0.123)
$\mathbf{r}_4$	0.933 (0.043)	0.836 (0.105)

[a] Average R-values (final 40 ns) along the 300 K and 400 K trajectories of each binding configuration starting from $\mathbf{r}_k$ with their respective standard deviation in parenthesis.

Table S2. Relative stability of the binding configurations for the ligand ABT-737.

	R-value [a] 300 K	R-value [a] 400 K
$\mathbf{r}_1$	0.969 (0.018)	0.821 (0.104)
$\mathbf{r}_2$	0.928 (0.052)	0.740 (0.099)
$\mathbf{r}_3$	0.909 (0.060)	0.622 (0.199)
$\mathbf{r}_4$	0.945 (0.055)	0.814 (0.116)
$\mathbf{r}_5$	0.980 (0.054)	0.331 (0.253)
$\mathbf{r}_6$	0.923 (0.068)	0.421 (0.204)
$\mathbf{r}_7$	0.906 (0.057)	0.628 (0.291)

[a] Average R-values (final 40 ns) along the 300 K and 400 K trajectories of each binding configuration starting from $\mathbf{r}_k$ with their respective standard deviation in parenthesis.

Table S3. Picking statistics ($r_1^W, r_2^W, r_3^W, r_4^W, r_1^A, r_2^A, r_3^A, r_4^A$).[a]

r_1^W											
**Window λ (Å)**	**Previous window's structure -> current window's structure R-value (RMSD in Å)**									**Cutoff**	**N**
	**1->1**	**1->2**	**1->3**	**2->1**	**2->2**	**2->3**	**3->1**	**3->2**	**3->3**		
**-2**	0.792 (4.40)	0.772 (4.36)	0.767 (4.57)	0.994 (2.22)	0.955 (2.27)	0.939 (2.37)	0.773 (4.22)	0.764 (4.01)	0.761 (4.08)	R:0.90	12045
**-1**	0.982 (1.39)	0.849 (3.22)	0.974 (1.93)	0.989 (1.95)	0.852 (3.70)	0.986 (2.37)	0.994 (1.56)	0.935 (2.91)	1.000 (1.33)	R:0.90	18315
**0**	1.000 (1.44)	0.936 (1.16)	0.980 (1.20)	NA	NA	NA	NA	NA	NA	R:0.90	447392
**1**	0.928 (1.70)	0.858 (1.98)	0.992 (1.53)	1.000 (1.15)	0.909 (2.36)	0.995 (1.71)	0.932 (2.05)	0.970 (1.83)	0.968 (1.88)	R:0.90	167806
**2**	0.930 (2.51)	0.928 (2.08)	0.821 (2.70)	0.954 (1.67)	0.856 (2.69)	0.955 (2.20)	0.910 (2.44)	0.946 (2.34)	0.868 (2.91)	R:0.90	8288
**3**	0.618 (4.88)	0.841 (1.99)	0.771 (3.46)	0.906 (3.15)	0.772 (2.82)	0.707 (3.21)	0.836 (4.05)	0.921 (1.83)	0.927 (2.77)	R:0.90	60
**4**	0.484 (6.70)	0.537 (5.64)	0.446 (5.79)	0.889 (5.29)	0.895 (4.12)	0.885 (4.02)	0.787 (4.94)	0.784 (3.99)	0.732 (5.07)	R:0.87	7
**5**	0.694 (4.79)	0.754 (2.49)	0.740 (2.16)	0.748 (3.98)	0.847 (4.10)	0.825 (3.68)	0.894 (3.40)	0.921 (3.42)	0.881 (3.85)	R:0.87	3
**6**	0.775 (5.87)	0.839 (4.27)	0.784 (4.51)	0.554 (5.46)	0.575 (5.13)	0.548 (5.07)	0.578 (4.82)	0.628 (4.50)	0.567 (4.29)	R:0.76	3
**7**	0.917 (4.81)	0.931 (6.21)	0.996 (4.52)	0.790 (4.88)	0.844 (4.72)	0.900 (2.41)	0.859 (4.97)	0.858 (4.87)	0.946 (3.04)	R:0.90	12
**8**	0.736 (4.09)	0.179 (5.37)	0.166 (4.95)	0.778 (3.42)	0.360 (3.94)	0.349 (5.28)	0.870 (3.86)	0.337 (3.17)	0.587 (3.53)	R:0.0 X:4.0	7
**10**	0.500 (4.59)	0.129 (4.50)	0.018 (6.62)	0.004 (4.68)	0.489 (5.27)	0.118 (5.71)	0.086 (7.05)	0.546 (5.67)	0.060 (4.87)	R:0.0 X:5.0	3
**12**	1.000 (1.67)	0.000 (4.19)	0.977 (2.35)	0.000 (5.03)	0.959 (3.13)	0.000 (6.16)	0.000 (7.27)	0.000 (6.54)	0.000 (7.25)	R:0.0 X:3.5	4
**14**	0.000 (6.61)	NA	NA	0.000 (4.95)	NA	NA	0.000 (6.33)	NA	NA	R:0.0 X:5.0	1
**16**	NA (5.93)	NA	NA	NA	NA	NA	NA	NA	NA	R:0.0 X:5.0	1
**18**	NA (3.83)	NA (3.18)	NA (3.42)	NA	NA	NA	NA	NA	NA	R:0.0 X:4.0	4
**20**	NA (60.62)	NA	NA	NA (59.73)	NA	NA	NA (61.56)	NA	NA	R:0.0 X:5.0	1
**22**	NA (5.39)	NA	NA	NA	NA	NA	NA	NA	NA	R:0.0 X:5.0	1
**24**	NA (4.60)	NA (4.01)	NA (3.12)	NA	NA	NA	NA	NA	NA	R:0.0 X:5.0	3
**26**	NA (6.50)	NA (4.03)	NA (6.85)	NA (4.85)	NA (8.08)	NA (4.67)	NA (5.53)	NA (6.12)	NA (5.97)	R:0.0 X:4.5	4
**28**	NA (3.22)	NA (8.20)	NA (3.20)	NA (9.08)	NA (2.83)	NA (8.04)	NA (2.77)	NA (8.36)	NA (3.56)	R:0.0 X:3.5	4
**30**	NA (6.31)	NA (9.73)	NA (9.33)	NA (9.01)	NA (4.78)	NA (5.32)	NA (4.91)	NA (8.61)	NA (9.40)	R:0.0 X:5.0	3

r_2^W											
**Window λ (Å)**	**Previous window's structure -> current window's structure R-value (RMSD in Å)**									**Cutoff**	**N**
	**1->1**	**1->2**	**1->3**	**2->1**	**2->2**	**2->3**	**3->1**	**3->2**	**3->3**		
**-2**	0.959 (3.01)	0.841 (2.11)	0.925 (2.86)	0.936 (2.89)	0.780 (2.16)	0.755 (3.31)	0.941 (2.55)	0.937 (2.35)	0.956 (2.13)	R:0.90	4303
**-1**	0.986 (1.26)	0.933 (1.33)	0.931 (2.01)	0.975 (1.69)	0.838 (1.38)	0.894 (2.63)	0.999 (1.28)	0.948 (1.39)	0.965 (1.55)	R:0.90	100615
**0**	0.965 (1.63)	0.987 (1.49)	0.986 (1.55)	NA	NA	NA	NA	NA	NA	R:0.90	318081
**1**	0.969 (2.22)	0.989 (1.19)	0.919 (3.52)	0.885 (2.01)	0.976 (1.72)	0.782 (4.29)	0.896 (2.76)	0.961 (1.74)	0.915 (3.95)	R:0.90	66563
**2**	0.961 (1.49)	0.880 (4.12)	0.788 (4.65)	0.930 (2.66)	0.934 (4.35)	0.927 (4.05)	0.862 (4.37)	0.988 (2.37)	0.982 (1.91)	R:0.90	11729
**3**	0.877 (4.14)	0.942 (2.87)	0.928 (3.89)	0.949 (2.53)	0.622 (6.21)	0.935 (3.56)	1.000 (1.31)	0.529 (7.01)	0.726 (5.39)	R:0.90	344
**4**	0.876 (1.97)	0.621 (6.70)	0.706 (5.82)	0.547 (6.95)	0.863 (3.39)	0.919 (3.66)	0.712 (5.08)	0.641 (5.81)	0.793 (2.85)	R:0.85	3
**5**	0.220 (10.99)	0.361 (8.69)	0.300 (9.65)	0.844 (5.87)	0.841 (4.53)	0.822 (6.19)	0.523 (7.17)	0.634 (5.39)	0.491 (7.69)	R:0.81	3
**6**	0.967 (2.68)	0.199 (5.35)	0.186 (6.17)	0.484 (5.81)	0.939 (2.83)	0.883 (2.70)	0.614 (6.22)	0.329 (5.58)	0.299 (5.93)	R:0.87	3
**7**	0.232 (8.44)	0.732 (3.16)	0.754 (3.69)	0.761 (6.90)	0.647 (5.01)	0.309 (6.66)	0.546 (6.88)	0.565 (6.37)	0.484 (7.27)	R:0.72	3
**8**	0.963 (4.02)	0.073 (9.42)	0.176 (10.13)	0.108 (7.84)	0.754 (4.12)	0.736 (4.36)	0.066 (7.11)	0.570 (6.19)	0.944 (2.82)	R:0.74	3
**10**	0.000 (9.26)	0.000 (9.21)	0.014 (8.54)	0.187 (7.13)	0.182 (7.11)	0.185 (6.52)	0.456 (4.67)	0.454 (4.65)	0.484 (4.91)	R:0.0 X:5.0	5
**12**	1.000 (2.66)	NA	NA	0.868 (3.39)	NA	NA	0.982 (3.48)	NA	NA	R:0.0 X:5.0	1
**14**	0.000 (5.06)	0.000 (5.58)	0.000 (5.00)	NA	NA	NA	NA	NA	NA	R:0.0 X:5.0	3
**16**	NA (4.61)	NA (7.15)	NA (6.90)	NA (5.93)	NA (5.87)	NA (5.63)	NA (6.12)	NA (5.27)	NA (5.63)	R:0.0 X:5.0	3
**18**	0.000 (9.01)	0.000 (5.69)	0.000 (10.26)	NA (2.69)	NA (10.63)	NA (5.17)	NA (4.05)	NA (9.96)	NA (6.23)	R:0.0 X:5.0	6
**20**	NA (9.63)	NA (6.77)	NA (7.40)	NA (4.05)	NA (12.78)	NA (13.79)	NA (11.18)	NA (2.56)	NA (5.14)	R:0.0 X:5.0	6
**22**	NA (12.07)	NA (12.82)	NA (12.27)	NA (7.02)	NA (6.33)	NA (7.08)	NA (1.85)	NA (2.68)	NA (2.54)	R:0.0 X:3.5	3
**24**	NA (5.59)	NA (5.46)	NA (6.38)	NA (5.68)	NA (4.82)	NA (4.69)	NA (5.69)	NA (5.95)	NA (5.91)	R:0.0 X:5.0	5
**26**	NA (2.88)	NA (3.59)	NA (7.59)	NA (5.16)	NA (7.08)	NA (5.32)	NA (7.37)	NA (8.49)	NA (3.23)	R:0.0 X:3.5	3
**28**	NA (65.77)	NA (59.91)	NA (4.63)	NA (66.98)	NA (60.47)	NA (4.65)	NA (62.26)	NA (62.61)	NA (7.00)	R:0.0 X:5.0	3
**30**	NA (67.53)	NA (68.71)	NA (68.75)	NA (61.86)	NA (61.89)	NA (61.82)	NA (4.34)	NA (3.00)	NA (4.50)	R:0.0 X:4.5	4

r_3^W											
**Window λ (Å)**	**Previous window's structure -> current window's structure R-value (RMSD in Å)**									**Cutoff**	**N**
	**1->1**	**1->2**	**1->3**	**2->1**	**2->2**	**2->3**	**3->1**	**3->2**	**3->3**		
**-2**	0.979 (1.48)	0.927 (1.99)	0.821 (2.33)	0.973 (1.00)	0.925 (1.35)	0.813 (1.80)	0.975 (1.61)	0.980 (1.97)	0.921 (2.08)	R:0.90	469
**-1**	0.940 (1.24)	0.904 (1.69)	0.953 (1.12)	0.960 (2.16)	0.988 (1.69)	0.934 (1.50)	0.961 (2.38)	0.951 (2.13)	0.929 (1.79)	R:0.90	70944
**0**	0.939 (1.92)	0.984 (1.99)	0.943 (1.20)	NA	NA	NA	NA	NA	NA	R:0.90	175722
**1**	0.772 (2.70)	0.817 (2.71)	0.951 (1.78)	0.819 (1.57)	0.894 (2.21)	0.879 (2.53)	0.940 (1.28)	0.964 (1.65)	0.913 (2.42)	R:0.90	53993
**2**	0.893 (1.62)	0.954 (1.75)	0.918 (1.81)	0.956 (1.86)	0.761 (2.53)	0.745 (2.72)	0.781 (3.48)	0.742 (2.64)	0.739 (3.03)	R:0.90	3852
**3**	0.707 (2.46)	0.617 (4.42)	0.681 (2.29)	0.618 (1.92)	0.906 (3.18)	0.592 (2.94)	0.921 (1.79)	0.778 (3.22)	0.917 (2.60)	R:0.90	157
**4**	0.926 (2.58)	0.924 (2.38)	0.945 (3.36)	0.595 (2.59)	0.601 (2.69)	0.704 (2.99)	0.880 (3.56)	0.836 (3.33)	0.849 (4.87)	R:0.90	4
**5**	0.780 (2.98)	0.783 (4.17)	0.923 (2.99)	0.618 (2.62)	0.528 (4.44)	0.919 (2.70)	0.485 (3.73)	0.495 (5.91)	0.727 (5.04)	R:0.77	4
**6**	0.971 (3.23)	0.917 (4.05)	0.918 (2.43)	0.478 (6.22)	0.440 (5.51)	0.546 (5.55)	0.472 (5.69)	0.476 (5.88)	0.561 (4.17)	R:0.90	20
**7**	0.704 (8.21)	0.914 (3.36)	0.728 (5.95)	0.578 (7.28)	0.819 (5.22)	0.650 (5.52)	0.618 (7.42)	0.916 (5.12)	0.653 (6.72)	R:0.70	4
**8**	0.839 (4.73)	0.828 (3.47)	0.821 (4.90)	0.492 (11.77)	0.470 (10.32)	0.324 (11.41)	0.706 (9.64)	0.544 (7.52)	0.556 (9.32)	R:0.81	7
**10**	0.769 (7.78)	0.871 (4.87)	0.424 (8.93)	0.710 (6.28)	0.789 (6.79)	0.419 (3.85)	0.728 (7.15)	0.684 (4.53)	0.761 (9.41)	R:0.75	3
**12**	0.376 (5.43)	0.086 (6.18)	0.044 (6.75)	0.293 (4.54)	0.141 (4.29)	0.065 (8.55)	0.172 (6.91)	0.057 (7.24)	0.043 (4.54)	R:0.7 X:4.5	6
**14**	0.093 (6.85)	0.872 (4.50)	0.388 (9.17)	0.056 (5.07)	0.379 (6.70)	0.080 (9.68)	0.000 (6.28)	0.001 (10.79)	0.999 (3.72)	R:0.0 X:5.0	4
**16**	NA (7.06)	NA (6.36)	NA (3.04)	0.000 (12.45)	0.000 (11.48)	0.000 (7.96)	0.000 (3.69)	0.000 (3.88)	0.000 (7.93)	R:0.0 X:4.5	3
**18**	NA (5.41)	NA	NA	NA (5.47)	NA	NA	0.000 (9.25)	NA	NA	R:0.0 X:5.0	1
**20**	NA (3.62)	NA (5.44)	NA	NA	NA	NA	NA	NA	NA	R:0.0 X:5.0	2
**22**	NA (3.89)	NA (6.62)	NA (5.73)	NA (3.98)	NA (4.35)	NA (4.21)	NA	NA	NA	R:0.0 X:4.5	3
**24**	NA (5.42)	NA (6.18)	NA (3.71)	NA (2.91)	NA (4.37)	NA (4.55)	NA (4.87)	NA (6.76)	NA (5.48)	R:0.0 X:4.5	5
**26**	NA (5.59)	NA (59.16)	NA (5.03)	NA (6.26)	NA (60.27)	NA (4.99)	NA (3.76)	NA (58.77)	NA (6.30)	R:0.0 X:5.0	4
**28**	NA (7.48)	NA (7.81)	NA (7.56)	NA (64.10)	NA (64.24)	NA (60.99)	NA (7.66)	NA (6.21)	NA (5.00)	R:0.0 X:5.0	3
**30**	NA (9.19)	NA (8.52)	NA (7.79)	NA (6.80)	NA (6.00)	NA (5.32)	NA (4.42)	NA (4.07)	NA (3.00)	R:0.0 X:5.0	3

r_4^W											
**Window λ (Å)**	**Previous window's structure -> current window's structure R-value (RMSD in Å)**									**Cutoff**	**N**
	**1->1**	**1->2**	**1->3**	**2->1**	**2->2**	**2->3**	**3->1**	**3->2**	**3->3**		
**-2**	0.894 (2.22)	0.936 (2.21)	0.865 (2.73)	0.988 (1.18)	0.697 (1.93)	0.928 (1.22)	0.915 (1.94)	0.894 (1.34)	0.896 (2.27)	R:0.90	10079
**-1**	0.846 (2.93)	0.790 (3.68)	0.943 (2.37)	0.743 (2.25)	0.935 (2.86)	0.890 (1.87)	0.921 (2.54)	0.958 (3.40)	0.935 (2.08)	R:0.90	98481
**0**	0.953 (1.50)	0.983 (1.50)	0.956 (1.27)	NA	NA	NA	NA	NA	NA	R:0.90	61569
**1**	0.990 (1.02)	0.938 (1.93)	0.952 (1.32)	0.893 (1.68)	0.781 (2.42)	0.864 (1.71)	0.998 (0.95)	0.932 (1.91)	0.843 (1.35)	R:0.90	22525
**2**	0.871 (4.11)	0.872 (4.57)	0.942 (2.01)	0.937 (3.56)	0.944 (3.89)	0.893 (2.55)	0.741 (4.38)	0.713 (4.83)	0.934 (1.76)	R:0.90	559
**3**	0.772 (5.08)	0.901 (2.88)	0.775 (5.67)	0.821 (5.28)	0.883 (3.46)	0.826 (5.67)	0.672 (3.09)	0.862 (3.01)	0.666 (8.58)	R:0.81	4
**4**	0.569 (11.47)	0.302 (7.44)	0.532 (5.36)	0.601 (10.35)	0.732 (6.84)	0.585 (4.79)	0.739 (4.18)	0.332 (8.86)	0.733 (6.16)	R:0.72	6
**5**	0.699 (8.42)	0.297 (11.07)	0.991 (5.97)	0.276 (6.31)	0.982 (1.49)	0.136 (9.43)	0.953 (3.28)	0.339 (6.44)	0.411 (7.63)	R:0.90	47
**6**	0.947 (4.72)	0.207 (8.44)	0.201 (7.49)	0.145 (6.51)	0.889 (4.07)	0.931 (2.35)	0.409 (6.97)	0.011 (11.67)	0.032 (10.23)	R:0.87	3
**7**	0.027 (9.98)	0.027 (9.46)	0.006 (10.58)	0.933 (5.91)	0.985 (5.26)	0.901 (6.36)	0.829 (7.13)	0.910 (5.64)	0.791 (7.76)	R:0.90	6
**8**	0.003 (3.76)	0.929 (1.76)	0.324 (5.05)	0.022 (5.13)	0.853 (3.58)	0.497 (6.28)	0.016 (4.60)	0.537 (3.19)	0.496 (3.78)	R:0.7 X:4.0	4
**10**	NA (4.10)	NA (6.46)	NA (2.47)	0.001 (5.06)	0.344 (6.95)	0.000 (4.62)	0.030 (3.45)	0.981 (3.27)	0.001 (4.25)	R:0.0 X:4.0	4
**12**	0.000 (5.12)	0.000 (6.41)	0.000 (5.81)	0.227 (8.01)	0.020 (8.65)	0.146 (7.06)	NA (4.81)	NA (5.38)	NA (6.96)	R:0.0 X:5.0	10
**14**	0.385 (7.30)	0.000 (3.83)	0.567 (3.95)	1.000 (8.05)	0.000 (7.70)	0.000 (7.23)	1.000 (3.49)	0.000 (6.96)	0.302 (6.48)	R:0.0 X:4.0	4
**16**	0.000 (10.69)	0.000 (7.52)	0.000 (4.98)	NA (5.24)	NA (4.53)	NA (7.92)	0.000 (7.67)	0.000 (4.13)	0.000 (6.88)	R:0.0 X:5.0	8
**18**	NA (10.98)	NA (12.16)	NA (8.77)	0.000 (7.35)	0.000 (8.97)	0.000 (5.99)	NA (4.16)	NA (4.97)	NA (4.65)	R:0.0 X:4.5	3
**20**	NA (7.16)	NA (6.61)	NA (4.92)	NA (7.79)	NA (7.48)	NA (3.93)	NA (4.59)	NA (4.21)	NA (6.91)	R:0.0 X:5.0	3
**22**	NA (5.06)	NA (5.76)	NA (4.97)	NA (3.02)	NA (5.63)	NA (5.36)	NA (6.71)	NA (9.38)	NA (10.00)	R:0.0 X:5.0	4
**24**	NA (4.95)	NA (6.04)	NA (6.10)	NA (5.62)	NA (3.17)	NA (4.76)	NA (7.53)	NA (4.51)	NA (7.39)	R:0.0 X:5.0	5
**26**	NA (3.99)	NA (3.21)	NA (2.51)	NA (6.06)	NA (7.31)	NA (6.55)	NA (2.07)	NA (3.99)	NA (3.65)	R:0.0 X:3.5	3
**28**	NA (59.06)	NA (57.69)	NA (58.09)	NA (59.79)	NA (58.45)	NA (58.81)	NA (60.32)	NA (58.86)	NA (59.28)	R:0.0 X:4.0	3
**30**	NA (6.98)	NA	NA	NA (6.29)	NA	NA	NA (5.78)	NA	NA	R:0.0 X:5.0	1

r_1^A											
**Window λ (Å)**	**Previous window's structure -> current window's structure R-value (RMSD in Å)**									**Cutoff**	**N**
	**1->1**	**1->2**	**1->3**	**2->1**	**2->2**	**2->3**	**3->1**	**3->2**	**3->3**		
**-2**	0.967 (1.76)	0.883 (2.24)	0.900 (1.98)	0.808 (3.50)	0.927 (2.73)	0.775 (3.13)	0.897 (2.50)	0.906 (1.92)	0.855 (2.28)	R:0.90	3
**-1**	0.741 (4.01)	0.999 (1.65)	0.806 (3.04)	0.916 (3.23)	0.922 (2.01)	0.881 (2.41)	0.891 (3.41)	0.915 (2.34)	0.918 (2.88)	R:0.90	1387
**0**	0.908 (1.96)	0.976 (1.62)	0.952 (1.70)	NA	NA	NA	NA	NA	NA	R:0.90	49408
**1**	0.774 (2.15)	0.856 (2.19)	0.822 (1.90)	0.908 (2.14)	0.941 (1.87)	0.955 (1.79)	0.927 (1.72)	0.976 (1.69)	0.987 (1.14)	R:0.90	111845
**2**	0.911 (1.58)	0.978 (1.96)	0.942 (2.39)	0.794 (1.83)	0.968 (1.88)	0.806 (2.59)	0.786 (2.55)	0.995 (1.80)	0.820 (3.13)	R:0.90	15015
**3**	0.954 (2.43)	0.919 (2.48)	0.814 (1.94)	0.676 (2.60)	0.698 (2.83)	0.846 (3.10)	0.766 (2.09)	0.758 (2.44)	1.000 (0.89)	R:0.90	3600
**4**	0.925 (2.02)	0.811 (5.04)	0.834 (4.47)	0.997 (1.09)	0.909 (4.79)	0.916 (4.12)	0.768 (2.50)	0.788 (6.14)	0.777 (5.24)	R:0.90	3176
**5**	0.860 (5.25)	0.881 (4.86)	0.909 (3.73)	0.891 (4.27)	0.996 (1.65)	0.934 (2.49)	0.990 (2.79)	1.000 (2.88)	0.994 (2.35)	R:0.90	92
**6**	0.905 (5.40)	0.948 (3.75)	0.910 (4.35)	0.712 (7.86)	0.769 (5.16)	0.703 (5.18)	0.819 (8.07)	0.840 (5.52)	0.835 (5.35)	R:0.90	43
**7**	0.994 (3.66)	0.981 (4.49)	0.986 (3.89)	0.980 (4.66)	0.971 (4.04)	0.974 (3.96)	0.985 (4.45)	0.978 (3.50)	0.980 (3.54)	R:0.90	454
**8**	0.994 (2.24)	0.967 (3.00)	0.993 (1.95)	1.000 (3.69)	0.996 (3.32)	1.000 (3.62)	1.000 (2.66)	0.996 (2.48)	0.998 (2.91)	R:0.90	3
**10**	0.482 (3.90)	0.368 (4.17)	0.275 (5.15)	0.359 (4.59)	0.266 (4.85)	0.227 (5.27)	0.458 (4.06)	0.321 (4.86)	0.290 (4.37)	R:0.7 X:4.0	5
**12**	0.072 (5.87)	0.060 (5.03)	0.500 (7.24)	0.082 (6.77)	0.029 (6.22)	0.568 (6.42)	0.043 (5.50)	0.016 (5.55)	0.705 (6.64)	R:0.7 X:5.0	4
**14**	0.750 (2.90)	0.000 (10.55)	0.000 (5.21)	0.000 (5.14)	0.254 (9.52)	0.006 (6.58)	0.165 (8.31)	0.001 (5.81)	0.000 (11.25)	R:0.0 X:5.0	11
**16**	0.042 (6.55)	0.021 (11.06)	0.000 (10.46)	0.029 (12.06)	0.931 (3.77)	0.928 (3.04)	1.000 (2.86)	0.000 (13.46)	0.000 (12.75)	R:0.0 X:3.5	3
**18**	0.000 (5.81)	0.000 (12.55)	0.000 (5.83)	0.000 (12.57)	0.004 (5.45)	0.000 (14.61)	0.000 (12.19)	0.104 (5.21)	0.000 (13.96)	R:0.0 X:5.0	4
**20**	NA (3.65)	NA (5.10)	NA	NA (11.58)	NA (13.20)	NA	NA (6.39)	NA (3.30)	NA	R:0.0 X:5.0	2
**22**	NA (7.68)	NA (7.06)	NA	NA (4.01)	NA (3.16)	NA	NA	NA	NA	R:0.0 X:5.0	2
**24**	NA (6.05)	NA	NA	NA (7.17)	NA	NA	NA	NA	NA	R:0.0 X:5.0	1
**26**	NA (4.48)	NA (4.31)	NA (3.96)	NA	NA	NA	NA	NA	NA	R:0.0 X:4.5	3
**28**	NA (2.94)	NA (6.45)	NA (7.26)	NA (4.92)	NA (5.97)	NA (7.19)	NA (5.49)	NA (4.78)	NA (4.21)	R:0.0 X:4.5	3
**30**	NA (4.30)	NA (2.54)	NA	NA (6.08)	NA (6.53)	NA	NA (6.87)	NA (7.22)	NA	R:0.0 X:5.0	2

r_2^A

Window λ (Å)	Previous window's structure -> current window's structure R-value (RMSD in Å)									Cutoff	N
	**1->1**	**1->2**	**1->3**	**2->1**	**2->2**	**2->3**	**3->1**	**3->2**	**3->3**		
**-2**	0.877 (2.18)	0.793 (3.44)	0.822 (3.23)	0.798 (3.08)	0.778 (3.39)	0.777 (3.98)	0.965 (1.40)	0.925 (2.53)	0.944 (2.24)	R:0.90	1346
**-1**	0.972 (2.44)	0.877 (3.02)	0.906 (2.47)	0.918 (2.52)	0.982 (1.98)	0.825 (3.28)	0.981 (1.22)	0.923 (2.84)	0.903 (2.12)	R:0.90	32840
**0**	0.976 (1.57)	0.923 (2.89)	0.973 (2.32)	NA	NA	NA	NA	NA	NA	R:0.90	70510
**1**	0.930 (2.14)	0.864 (2.69)	0.962 (1.86)	0.817 (3.76)	0.882 (2.65)	0.897 (2.92)	0.901 (2.41)	0.924 (1.96)	0.975 (1.83)	R:0.90	2712
**2**	0.873 (2.59)	0.812 (3.93)	0.832 (3.20)	0.701 (2.92)	0.856 (2.65)	0.993 (1.87)	0.796 (2.87)	0.873 (3.29)	0.905 (2.67)	R:0.87	3
**3**	0.510 (4.97)	0.445 (5.58)	0.797 (4.60)	0.752 (3.50)	0.796 (3.44)	0.603 (5.02)	0.796 (4.07)	0.667 (4.64)	0.600 (5.12)	R:0.79	3
**4**	0.412 (8.68)	0.410 (8.09)	0.376 (8.16)	0.282 (8.35)	0.302 (7.77)	0.308 (7.67)	0.923 (3.54)	0.886 (4.03)	0.884 (3.22)	R:0.88	3
**5**	0.384 (6.29)	0.621 (4.28)	0.490 (5.04)	0.434 (4.50)	0.763 (4.20)	0.624 (5.24)	0.443 (5.21)	0.694 (3.71)	0.624 (4.53)	R:0.7 X:4.5	4
**6**	0.370 (6.08)	0.932 (4.37)	0.648 (5.19)	0.847 (2.88)	0.406 (5.67)	0.905 (3.35)	0.903 (2.35)	0.391 (5.73)	0.999 (2.87)	R:0.90	31
**7**	0.646 (6.16)	0.270 (7.07)	0.842 (3.68)	0.910 (3.76)	0.926 (5.29)	0.632 (4.99)	0.463 (5.26)	0.530 (5.45)	0.999 (1.89)	R:0.90	15
**8**	0.696 (4.12)	0.905 (3.13)	0.679 (4.21)	0.908 (5.05)	0.702 (5.00)	0.951 (3.31)	0.561 (4.38)	0.433 (5.67)	0.480 (4.70)	R:0.90	17
**10**	0.401 (7.53)	0.486 (4.89)	0.780 (5.07)	0.782 (5.33)	0.789 (5.54)	0.472 (7.07)	0.367 (7.55)	0.443 (4.99)	0.781 (6.16)	R:0.78	3
**12**	0.793 (6.48)	0.800 (5.80)	0.791 (6.74)	0.368 (7.45)	0.311 (7.57)	0.470 (8.29)	0.359 (6.91)	0.423 (6.85)	0.400 (7.25)	R:0.79	3
**14**	0.623 (5.10)	0.608 (4.43)	0.558 (5.86)	0.408 (5.08)	0.459 (4.20)	0.466 (5.22)	0.431 (4.30)	0.447 (5.03)	0.449 (4.25)	R:0.7 X:3.5	3
**16**	0.417 (5.48)	0.060 (7.13)	0.043 (7.24)	0.222 (6.61)	0.062 (7.78)	0.049 (7.36)	0.236 (6.43)	0.092 (5.44)	0.100 (5.79)	R:0.7 X:5.0	3
**18**	0.980 (2.91)	0.288 (3.99)	0.057 (8.99)	0.000 (9.19)	0.000 (8.43)	0.471 (3.57)	0.060 (9.27)	0.000 (8.75)	0.638 (2.06)	R:0.0 X:4.0	3
**20**	0.132 (5.75)	NA	NA	0.000 (6.62)	NA	NA	0.139 (12.72)	NA	NA	R:0.0 X:5.0	1
**22**	0.921 (3.85)	NA	NA	NA	NA	NA	NA	NA	NA	R:0.0 X:5.0	1
**24**	0.999 (4.66)	0.406 (3.65)	NA	NA	NA	NA	NA	NA	NA	R:0.0 X:5.0	2
**26**	0.000 (6.09)	NA	NA	NA (6.82)	NA	NA	NA	NA	NA	R:0.0 X:5.0	1
**28**	NA (4.94)	NA	NA	NA	NA	NA	NA	NA	NA	R:0.0 X:5.0	1
**30**	NA (5.18)	NA (3.32)	NA	NA	NA	NA	NA	NA	NA	R:0.0 X:5.0	2

r_3^A											
**Window λ (Å)**	**Previous window's structure -> current window's structure R-value (RMSD in Å)**									**Cutoff**	**N**
	**1->1**	**1->2**	**1->3**	**2->1**	**2->2**	**2->3**	**3->1**	**3->2**	**3->3**		
**-2**	0.908 (1.18)	0.884 (2.19)	0.991 (1.21)	0.892 (3.51)	0.810 (4.06)	0.925 (3.56)	0.832 (1.99)	0.943 (1.84)	0.861 (2.14)	R:0.90	1042
**-1**	0.843 (3.01)	0.799 (3.25)	0.883 (3.26)	0.890 (3.22)	0.896 (2.84)	0.837 (3.58)	0.949 (3.43)	0.999 (1.07)	0.916 (3.90)	R:0.90	18379
**0**	0.919 (2.17)	0.939 (2.32)	0.909 (2.66)	NA	NA	NA	NA	NA	NA	R:0.90	10305
**1**	0.919 (1.99)	0.825 (3.20)	0.973 (1.42)	0.810 (2.53)	0.821 (2.92)	0.817 (2.52)	0.744 (3.47)	0.941 (2.31)	0.800 (3.24)	R:0.90	33044
**2**	0.974 (1.85)	0.911 (1.69)	0.802 (3.45)	0.749 (3.35)	0.688 (3.36)	0.909 (2.24)	0.935 (2.19)	0.882 (1.80)	0.795 (3.30)	R:0.90	618
**3**	0.787 (3.00)	0.753 (2.54)	0.713 (2.98)	0.921 (2.44)	0.986 (1.85)	0.958 (2.22)	0.532 (4.10)	0.511 (4.11)	0.501 (3.90)	R:0.90	23
**4**	0.635 (5.16)	0.711 (4.75)	0.704 (4.39)	0.830 (4.25)	0.819 (4.00)	0.804 (3.83)	0.813 (4.24)	0.841 (3.52)	0.833 (3.30)	R:0.82	3
**5**	0.827 (4.47)	0.846 (5.99)	0.773 (7.58)	0.460 (7.36)	0.382 (9.13)	0.380 (10.84)	0.490 (7.08)	0.401 (8.57)	0.303 (10.52)	R:0.77	3
**6**	0.719 (12.81)	0.725 (12.21)	0.708 (12.66)	0.915 (11.92)	0.903 (11.36)	0.901 (12.15)	0.586 (8.16)	0.593 (7.44)	0.518 (8.35)	R:0.90	3
**7**	0.790 (4.70)	0.881 (7.23)	0.912 (3.98)	0.707 (4.30)	0.935 (7.22)	0.711 (3.44)	0.908 (2.85)	0.847 (5.49)	0.879 (3.14)	R:0.90	5
**8**	0.550 (4.33)	0.890 (3.39)	0.959 (3.37)	0.920 (4.02)	0.500 (6.71)	0.575 (7.02)	0.698 (5.10)	0.602 (3.76)	0.765 (3.35)	R:0.89	3
**10**	0.000 (7.21)	0.074 (5.31)	0.953 (3.99)	0.000 (5.24)	0.204 (6.81)	0.561 (4.30)	0.000 (5.68)	0.145 (6.68)	0.423 (4.46)	R:0.0 X:5.0	8
**12**	NA (9.24)	NA (4.85)	NA (7.60)	0.000 (4.63)	0.000 (8.90)	0.629 (6.33)	0.077 (6.72)	0.000 (9.96)	0.161 (5.91)	R:0.0 X:5.0	4
**14**	0.571 (7.33)	0.233 (5.04)	NA	NA (11.11)	NA (12.64)	NA	0.123 (4.53)	0.001 (8.70)	NA	R:0.0 X:5.0	2
**16**	0.369 (10.35)	0.567 (3.57)	NA	0.857 (5.80)	0.916 (6.91)	NA	NA	NA	NA	R:0.0 X:5.0	2
**18**	0.495 (3.10)	0.000 (8.47)	0.636 (7.85)	0.493 (8.46)	0.031 (2.57)	0.658 (2.72)	NA	NA	NA	R:0.0 X:3.5	3
**20**	0.052 (4.93)	0.002 (4.38)	NA	NA (11.25)	NA (10.21)	NA	0.921 (10.78)	0.796 (9.83)	NA	R:0.0 X:5.0	2
**22**	0.000 (6.62)	NA	NA	NA (6.38)	NA	NA	NA	NA	NA	R:0.0 X:5.0	1
**24**	NA (4.12)	NA (3.76)	NA (3.77)	NA	NA	NA	NA	NA	NA	R:0.0 X:4.0	3
**26**	NA (4.48)	NA (6.02)	NA (4.42)	NA (3.28)	NA (5.99)	NA (4.65)	NA (6.65)	NA (3.64)	NA (3.59)	R:0.0 X:3.5	3
**28**	NA (3.01)	NA (4.64)	NA (8.38)	NA (8.28)	NA (7.07)	NA (4.08)	NA (7.68)	NA (6.17)	NA (6.14)	R:0.0 X:4.0	3
**30**	NA (5.47)	NA	NA	NA (5.90)	NA	NA	NA (8.91)	NA	NA	R:0.0 X:5.0	1

r_4^A											
**Window λ (Å)**	**Previous window's structure -> current window's structure R-value (RMSD in Å)**									**Cutoff**	**N**
	**1->1**	**1->2**	**1->3**	**2->1**	**2->2**	**2->3**	**3->1**	**3->2**	**3->3**		
**-2**	0.945 (1.90)	0.878 (2.98)	0.877 (3.96)	0.821 (3.64)	0.901 (2.50)	0.937 (1.92)	0.794 (2.55)	0.902 (2.47)	0.788 (3.17)	R:0.90	6
**-1**	0.936 (2.14)	0.900 (2.98)	0.938 (2.39)	0.935 (1.43)	0.870 (3.56)	0.892 (1.77)	0.879 (2.85)	0.961 (2.28)	0.829 (2.58)	R:0.90	14724
**0**	0.946 (2.04)	0.930 (2.12)	0.921 (2.21)	NA	NA	NA	NA	NA	NA	R:0.90	35698
**1**	0.703 (2.07)	0.850 (3.15)	0.879 (2.27)	0.928 (1.69)	0.860 (3.55)	0.956 (1.59)	0.771 (2.72)	0.960 (2.63)	0.841 (2.87)	R:0.90	17187
**2**	0.805 (2.91)	0.918 (2.03)	0.788 (2.61)	0.679 (4.45)	0.674 (3.52)	0.743 (3.88)	0.936 (2.61)	0.797 (2.62)	0.908 (2.34)	R:0.90	109
**3**	0.910 (1.64)	0.903 (2.50)	0.912 (2.48)	0.747 (3.44)	0.819 (3.89)	0.664 (4.47)	0.829 (2.48)	0.759 (3.09)	0.792 (3.62)	R:0.90	10
**4**	0.827 (3.19)	0.811 (2.94)	0.836 (3.74)	0.907 (2.54)	0.918 (1.56)	0.913 (2.56)	0.698 (3.79)	0.807 (2.95)	0.887 (3.80)	R:0.90	6
**5**	0.759 (4.45)	0.863 (4.31)	0.735 (3.80)	0.914 (4.58)	0.927 (4.95)	0.917 (3.22)	0.794 (4.55)	0.780 (4.95)	0.785 (3.31)	R:0.90	15
**6**	0.973 (3.05)	0.934 (5.27)	0.921 (4.41)	0.927 (3.98)	0.836 (6.16)	0.899 (3.48)	0.934 (2.91)	0.803 (4.40)	0.835 (5.46)	R:0.90	7
**7**	0.779 (5.75)	0.825 (3.46)	0.785 (7.37)	0.828 (4.66)	0.708 (5.69)	0.815 (6.91)	0.784 (5.27)	0.688 (4.02)	0.703 (5.99)	R:0.81	3
**8**	0.575 (10.13)	0.752 (6.96)	0.697 (7.16)	0.568 (11.91)	0.664 (3.28)	0.623 (8.29)	0.744 (7.40)	0.627 (6.76)	0.993 (2.50)	R:0.74	3
**10**	0.977 (5.16)	0.903 (4.77)	0.990 (5.04)	0.691 (7.96)	0.780 (9.05)	0.714 (8.76)	0.661 (5.31)	0.811 (5.99)	0.747 (5.45)	R:0.90	3
**12**	0.325 (5.36)	0.759 (7.67)	0.244 (6.50)	0.392 (5.73)	0.708 (6.90)	0.059 (7.46)	0.162 (5.69)	0.628 (9.17)	0.139 (5.81)	R:0.7 X:5.0	10
**14**	0.027 (7.22)	0.022 (7.39)	0.000 (8.31)	0.007 (10.32)	0.137 (10.21)	0.248 (11.43)	0.257 (3.00)	0.998 (2.23)	1.000 (2.85)	R:0.0 X:3.0	4
**16**	0.000 (4.19)	NA	NA	0.019 (4.53)	NA	NA	0.488 (5.20)	NA	NA	R:0.0 X:5.0	1
**18**	NA (4.88)	NA (4.76)	NA (2.63)	NA	NA	NA	NA	NA	NA	R:0.0 X:5.0	4
**20**	NA (3.65)	NA (5.10)	NA	NA (6.97)	NA (5.20)	NA	NA (6.39)	NA (3.30)	NA	R:0.0 X:5.0	2
**22**	NA (4.28)	NA (7.68)	NA (7.06)	NA (7.36)	NA (4.01)	NA (3.16)	NA	NA	NA	R:0.0 X:4.0	3
**24**	NA (3.64)	NA (9.73)	NA (4.53)	NA (11.28)	NA (4.29)	NA (11.98)	NA (10.45)	NA (5.10)	NA (11.18)	R:0.0 X:4.5	4
**26**	NA (5.91)	NA (11.60)	NA (5.31)	NA (14.68)	NA (4.69)	NA (11.76)	NA (5.51)	NA (12.30)	NA (6.19)	R:0.0 X:5.0	4
**28**	NA (5.21)	NA (13.66)	NA (14.17)	NA (15.96)	NA (3.06)	NA (3.38)	NA (9.67)	NA (10.02)	NA (10.75)	R:0.0 X:5.0	3
**30**	NA (5.64)	NA (3.46)	NA (2.74)	NA (15.15)	NA (16.53)	NA (14.73)	NA (15.49)	NA (16.87)	NA (15.41)	R:0.0 X:5.0	3

[a] Listed are the statistics for the pathways starting from $\mathbf{q}_1$-$\mathbf{q}_4$ for the WEHI-539 and ABT-737 ligands. The algorithm starts at window where λ = 0 Å with the structure $\mathbf{q}_k$, picks 2 other similar structures for this window, and then moves to windows -1 and -1, picking 3 representative structures that are similar to $\mathbf{q}_k$ and the other two picked from the λ=0 window. Then, the process is repeated for the windows -2 and +2, picking structures similar to those of the preceding windows, i.e. those from -1 and +1, respectively. Shown are the similarities between the picked structures from the preceding window to the current window, in terms of the R-value and the RMSD of the ligand. In case no such comparison exists (e.g. there were fewer structures in the previous window, or fewer structure in the current window), an "NA" is shown instead. Finally, the cutoff used is listed, where "R:" corresponds to the R-value cutoff and "X:" to the RMSD based cutoff, where matching structures only have to fulfill one of the criteria and the number of matching structures is listed in the final column "N". In case the number of contacts is less than 25, the R-value cutoff is no longer used (in these cases, R:0.0 is set).

Table S4. Free energy obtained for ligand WEHI-539 in configuration q_1.[a]

Start (ns)	End (ns)	ΔG	σ	ε	ΔG_b^0	V (nm^3)
40	250	16.33	0.01	0.01	-11.85	0.905
50	250	16.22	0.01	0.01	-11.72	0.878
60	250	16.06	0.01	0.01	-11.56	0.875
70	250	15.94	0.01	0.01	-11.47	0.915
80	250	15.90	0.01	0.01	-11.42	0.905
90	250	15.89	0.01	0.01	-11.40	0.877
100	250	15.90	0.01	0.01	-11.37	0.831
110	250	15.86	0.01	0.01	-11.33	0.832
120	250	16.04	0.01	0.01	-11.50	0.823
130	250	16.10	0.01	0.01	-11.55	0.807
140	250	16.17	0.01	0.02	-11.60	0.786
150	250	16.16	0.01	0.02	-11.63	0.836
160	250	16.26	0.01	0.02	-11.72	0.825
170	250	16.41	0.01	0.02	-11.87	0.817
180	250	16.49	0.01	0.02	-11.97	0.846
190	250	16.48	0.01	0.02	-11.98	0.865
200	250	16.88	0.01	0.02	-12.36	0.841
210	250	17.14	0.01	0.02	-12.60	0.815

[a] All WHAM calculations were executed using the same parameters as the calculations used in the main text (i.e. a Δλ of 0.05 Å, a tolerance of $1e^{-8}$ and with 1000 bootstraps). ΔG is the average PMF over the final 50 bins (2.5 Å), with σ its standard deviation. ε corresponds to the average error (via bootstrapping) taken over the same range. ΔG_b^0 is the standard binding free energy calculated using ΔG and the corresponding sample COMs during the start and end range of the simulation following Eq. S7 and V corresponds to the weighted sampled volume as calculated part of the equation.

Table S5. Free energy obtained for ligand WEHI-539 in configuration q_2. [a]

Start (ns)	End (ns)	ΔG	σ	ε	ΔG_b^0	V (nm^3)
40	250	17.31	0.01	0.01	-12.63	0.648
50	250	17.24	0.01	0.01	-12.56	0.645
60	250	17.14	0.01	0.01	-12.46	0.652
70	250	17.09	0.01	0.01	-12.42	0.652
80	250	17.02	0.01	0.01	-12.35	0.649
90	250	17.01	0.01	0.01	-12.33	0.647
100	250	16.98	0.01	0.01	-12.31	0.650
110	250	16.92	0.01	0.01	-12.25	0.659
120	250	16.86	0.01	0.02	-12.19	0.661
130	250	16.94	0.01	0.02	-12.25	0.637
140	250	16.99	0.01	0.02	-12.31	0.647
150	250	16.89	0.01	0.02	-12.21	0.641
160	250	16.95	0.01	0.02	-12.25	0.629
170	250	16.83	0.01	0.02	-12.11	0.601
180	250	16.75	0.01	0.02	-12.00	0.575
190	250	16.67	0.01	0.02	-11.89	0.546
200	250	16.56	0.01	0.02	-11.78	0.545
210	250	16.38	0.01	0.03	-11.59	0.539

[a] All WHAM calculations were executed using the same parameters as the calculations used in the main text (i.e. a Δλ of 0.05 Å, a tolerance of $1e^{-8}$ and with 1000 bootstraps). ΔG is the average PMF over the final 50 bins (2.5 Å), with σ its standard deviation. ε corresponds to the average error (via bootstrapping) taken over the same range. ΔG_b^0 is the standard binding free energy calculated using ΔG and the corresponding sample COMs during the start and end range of the simulation following Eq. S7 and V corresponds to the weighted sampled volume as calculated part of the equation.

Table S6. Free energy obtained for ligand WEHI-539 in configuration q3. [a]

Start (ns)	End (ns)	ΔG	σ	ε	ΔG_b^0	V (nm^3)
40	250	21.68	0.01	0.01	-17.26	1.009
50	250	21.78	0.00	0.01	-17.36	0.993
60	250	21.89	0.01	0.01	-17.45	0.971
70	250	21.89	0.01	0.01	-17.44	0.955
80	250	21.89	0.01	0.01	-17.45	0.968
90	250	21.90	0.01	0.01	-17.44	0.936
100	250	21.92	0.01	0.01	-17.47	0.940
110	250	21.93	0.01	0.01	-17.46	0.921
120	250	21.98	0.01	0.01	-17.50	0.914
130	250	22.03	0.01	0.02	-17.54	0.886
140	250	22.26	0.01	0.02	-17.76	0.870
150	250	22.44	0.01	0.02	-17.95	0.888
160	250	22.54	0.01	0.02	-18.02	0.844
170	250	22.75	0.01	0.02	-18.23	0.841
180	250	22.84	0.01	0.02	-18.32	0.844
190	250	22.91	0.01	0.02	-18.34	0.781
200	250	22.96	0.01	0.02	-18.38	0.763
210	250	22.83	0.01	0.03	-18.21	0.714

[a] All WHAM calculations were executed using the same parameters as the calculations used in the main text (i.e. a Δλ of 0.05 Å, a tolerance of $1e^{-8}$ and with 1000 bootstraps). ΔG is the average PMF over the final 50 bins (2.5 Å), with σ its standard deviation. ε corresponds to the average error (via bootstrapping) taken over the same range. ΔG_b^0 is the standard binding free energy calculated using ΔG and the corresponding sample COMs during the start and end range of the simulation following Eq. S7 and V corresponds to the weighted sampled volume as calculated part of the equation.

Table S7. Free energy obtained for ligand WEHI-539 in configuration q_4.[a]

Start (ns)	End (ns)	ΔG	σ	ε	ΔG_b^0	V (nm^3)
40	250	19.44	0.01	0.01	-15.19	1.348
50	250	19.60	0.01	0.01	-15.39	1.407
60	250	19.70	0.01	0.01	-15.45	1.340
70	250	19.83	0.01	0.01	-15.55	1.268
80	250	20.03	0.01	0.01	-15.72	1.203
90	250	20.14	0.01	0.01	-15.82	1.189
100	250	20.25	0.01	0.01	-15.89	1.112
110	250	20.40	0.01	0.01	-16.03	1.099
120	250	20.35	0.01	0.02	-16.03	1.194
130	250	20.30	0.01	0.02	-15.97	1.168
140	250	20.23	0.01	0.02	-15.95	1.257
150	250	20.26	0.01	0.02	-16.00	1.302
160	250	20.27	0.01	0.02	-16.00	1.287
170	250	20.12	0.01	0.02	-15.93	1.478
180	250	20.11	0.01	0.02	-15.86	1.332
190	250	20.07	0.01	0.02	-15.72	1.138
200	250	19.92	0.02	0.02	-15.47	0.957
210	250	19.50	0.02	0.03	-14.95	0.808

[a] All WHAM calculations were executed using the same parameters as the calculations used in the main text (i.e. a Δλ of 0.05 Å, a tolerance of 1e^{-8} and with 1000 bootstraps). ΔG is the average PMF over the final 50 bins (2.5 Å), with σ its standard deviation. ε corresponds to the average error (via bootstrapping) taken over the same range. ΔG_b^0 is the standard binding free energy calculated using ΔG and the corresponding sample COMs during the start and end range of the simulation following Eq. S7 and V corresponds to the weighted sampled volume as calculated part of the equation.

Table S8. Free energy obtained for ligand ABT-737 in configuration q_1.[a]

Start (ns)	End (ns)	ΔG	σ	ε	ΔG_b^0	V (nm^3)
40	250	19.88	0.01	0.01	-15.43	0.954
50	250	19.84	0.01	0.01	-15.36	0.906
60	250	19.84	0.01	0.01	-15.35	0.890
70	250	19.77	0.01	0.01	-15.25	0.852
80	250	19.83	0.01	0.01	-15.30	0.830
90	250	19.78	0.01	0.01	-15.22	0.792
100	250	19.72	0.01	0.01	-15.15	0.783
110	250	19.69	0.01	0.01	-15.14	0.800
120	250	19.63	0.01	0.01	-15.07	0.794
130	250	19.68	0.01	0.02	-15.09	0.758
140	250	19.85	0.01	0.02	-15.22	0.711
150	250	20.09	0.01	0.02	-15.45	0.696
160	250	20.25	0.01	0.02	-15.57	0.646
170	250	20.44	0.01	0.02	-15.75	0.638
180	250	20.78	0.01	0.02	-16.1	0.645
190	250	21.06	0.01	0.02	-16.38	0.654
200	250	21.28	0.02	0.02	-16.67	0.722
210	250	21.67	0.02	0.03	-17.03	0.687

[a] All WHAM calculations were executed using the same parameters as the calculations used in the main text (i.e. a Δλ of 0.05 Å, a tolerance of $1e^{-8}$ and with 1000 bootstraps). ΔG is the average PMF over the final 50 bins (2.5 Å), with σ its standard deviation. ε corresponds to the average error (via bootstrapping) taken over the same range. ΔG_b^0 is the standard binding free energy calculated using ΔG and the corresponding sample COMs during the start and end range of the simulation following Eq. S7 and V corresponds to the weighted sampled volume as calculated part of the equation.

Table S9. Free energy obtained for ligand ABT-737 in configuration q_2. [a]

Start (ns)	End (ns)	ΔG	σ	ε	ΔG_b^0	V (nm^3)
40	250	20.22	0.01	0.01	-15.86	1.105
50	250	20.20	0.01	0.01	-15.84	1.117
60	250	20.12	0.01	0.01	-15.78	1.146
70	250	20.06	0.01	0.01	-15.72	1.139
80	250	19.94	0.01	0.01	-15.59	1.113
90	250	19.95	0.01	0.01	-15.60	1.128
100	250	20.04	0.01	0.01	-15.68	1.106
110	250	20.12	0.01	0.01	-15.74	1.078
120	250	20.30	0.01	0.01	-15.91	1.042
130	250	20.38	0.01	0.02	-15.99	1.053
140	250	20.44	0.01	0.02	-16.04	1.019
150	250	20.55	0.01	0.02	-16.12	0.993
160	250	20.65	0.01	0.02	-16.17	0.918
170	250	20.68	0.01	0.02	-16.19	0.891
180	250	20.70	0.01	0.02	-16.19	0.855
190	250	20.95	0.01	0.02	-16.47	0.900
200	250	20.97	0.01	0.02	-16.38	0.754
210	250	20.63	0.01	0.03	-16.00	0.707

[a] All WHAM calculations were executed using the same parameters as the calculations used in the main text (i.e. a Δλ of 0.05 Å, a tolerance of $1e^{-8}$ and with 1000 bootstraps). ΔG is the average PMF over the final 50 bins (2.5 Å), with σ its standard deviation. ε corresponds to the average error (via bootstrapping) taken over the same range. ΔG_b^0 is the standard binding free energy calculated using ΔG and the corresponding sample COMs during the start and end range of the simulation following Eq. S7 and V corresponds to the weighted sampled volume as calculated part of the equation.

Table S10. Free energy obtained for ligand ABT-737 in configuration q3. [a]

Start (ns)	End (ns)	ΔG	σ	ε	ΔG_b^0	V (nm^3)
40	250	16.56	0.01	0.01	-12.10	0.934
50	250	16.47	0.01	0.01	-12.00	0.911
60	250	16.31	0.01	0.01	-11.83	0.912
70	250	16.27	0.01	0.01	-11.81	0.942
80	250	16.20	0.01	0.01	-11.72	0.903
90	250	16.21	0.01	0.01	-11.71	0.877
100	250	16.25	0.01	0.01	-11.77	0.896
110	250	16.10	0.01	0.01	-11.60	0.884
120	250	16.05	0.01	0.01	-11.57	0.906
130	250	15.90	0.01	0.02	-11.42	0.911
140	250	16.10	0.01	0.02	-11.60	0.879
150	250	16.07	0.01	0.02	-11.56	0.857
160	250	16.05	0.01	0.02	-11.50	0.811
170	250	15.97	0.01	0.02	-11.39	0.776
180	250	16.12	0.01	0.02	-11.62	0.877
190	250	16.14	0.01	0.02	-11.57	0.784
200	250	16.35	0.01	0.02	-11.80	0.813
210	250	16.09	0.01	0.03	-11.59	0.877

[a] All WHAM calculations were executed using the same parameters as the calculations used in the main text (i.e. a Δλ of 0.05 Å, a tolerance of $1e^{-8}$ and with 1000 bootstraps). ΔG is the average PMF over the final 50 bins (2.5 Å), with σ its standard deviation. ε corresponds to the average error (via bootstrapping) taken over the same range. ΔG_b^0 is the standard binding free energy calculated using ΔG and the corresponding sample COMs during the start and end range of the simulation following Eq. S7 and V corresponds to the weighted sampled volume as calculated part of the equation.

Table S11. Free energy obtained for ligand ABT-737 in configuration q_4.[a]

Start (ns)	End (ns)	ΔG	σ	ε	ΔG_b^0	V (nm^3)
40	250	20.27	0.01	0.01	-15.67	0.740
50	250	20.23	0.01	0.01	-15.61	0.727
60	250	20.14	0.01	0.01	-15.53	0.717
70	250	20.04	0.01	0.01	-15.43	0.727
80	250	19.87	0.01	0.01	-15.25	0.712
90	250	19.86	0.01	0.01	-15.22	0.698
100	250	19.70	0.01	0.01	-15.06	0.691
110	250	19.49	0.01	0.01	-14.86	0.697
120	250	19.46	0.01	0.02	-14.83	0.704
130	250	19.40	0.01	0.02	-14.77	0.703
140	250	19.30	0.01	0.02	-14.70	0.742
150	250	19.11	0.01	0.02	-14.50	0.725
160	250	19.09	0.01	0.02	-14.44	0.681
170	250	19.05	0.01	0.02	-14.34	0.623
180	250	18.95	0.01	0.02	-14.20	0.577
190	250	19.01	0.01	0.02	-14.24	0.557
200	250	18.97	0.01	0.02	-14.17	0.532
210	250	18.93	0.01	0.03	-14.13	0.533

[a] All WHAM calculations were executed using the same parameters as the calculations used in the main text (i.e. a Δλ of 0.05 Å, a tolerance of $1e^{-8}$ and with 1000 bootstraps). ΔG is the average PMF over the final 50 bins (2.5 Å), with σ its standard deviation. ε corresponds to the average error (via bootstrapping) taken over the same range. ΔG_b^0 is the standard binding free energy calculated using ΔG and the corresponding sample COMs during the start and end range of the simulation following Eq. S7 and V corresponds to the weighted sampled volume as calculated part of the equation.

Table S12. Window characteristics. [a]

Lower λ'_l (Å)	Center λ'_c (Å)	Upper λ'_u (Å)	US window center λ (Å)	Force constant (kcal/mol/Å^2)
-2.50	-2.00	-1.50	-2.00	5.00
-1.50	-1.00	-0.50	-1.00	5.00
-0.50	0.00	0.50	0.00	5.00
0.50	1.00	1.50	1.00	5.00
1.50	2.00	2.50	2.00	5.00
2.50	3.00	3.50	3.00	5.00
3.50	4.00	4.50	4.00	5.00
4.50	5.00	5.50	5.00	5.00
5.50	6.00	6.50	6.00	5.00
6.50	7.00	7.50	7.00	4.25
7.50	8.00	9.00	8.25	2.75
9.00	10.00	11.00	10.00	2.00
11.00	12.00	13.00	12.00	1.75
13.00	14.00	15.00	14.00	1.75
15.00	16.00	17.00	16.00	1.75
17.00	18.00	19.00	18.00	1.75
19.00	20.00	21.00	20.00	1.75
21.00	22.00	23.00	22.00	1.75
23.00	24.00	25.00	24.00	1.75
25.00	26.00	27.00	26.00	1.75
27.00	28.00	29.00	28.00	1.75
29.00	30.00	31.00	30.00	1.75

[a] Window parameters used during the path generation and US simulations. λ'_l, λ'_u and λ'_c are the lower and upper ranges and the center of each window during the picking stage, respectively. The center of the window as used during the simulations is shown in the fourth column. This deviates slightly from the picking center in some cases, to smooth out the distances between the window centers. The force constant as used by the US simulations are shown in the last column. The window at $\lambda'_c = 0$ Å corresponds to the initial window, where the reference structure corresponds to one of $\boldsymbol{r}_k^W$ or $\boldsymbol{r}_k^A$ (with k ranging from 1 – 4). For $\boldsymbol{r}_1^W$, the final three windows were omitted, as they produced structures that would have the ligand and protein approaching each other, due to periodic boundary conditions.

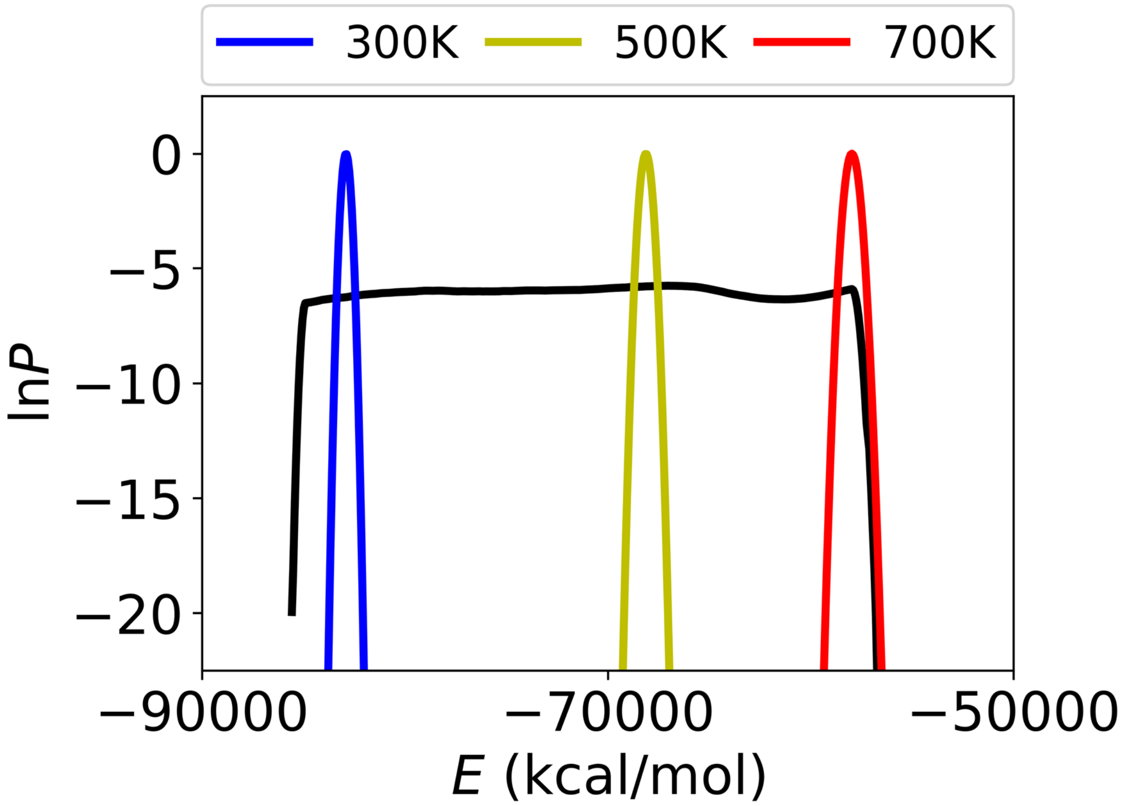

Figure S1. WEHI-539 McMD potential energy distribution. Potential energy probability distribution ($P_{McMD}(E)$) as sampled during the production run. Also shown are the reweighing canonical distributions ($P_c(E, T)$) at 300 K, 500 K and 700 K in blue, yellow and red, respectively.

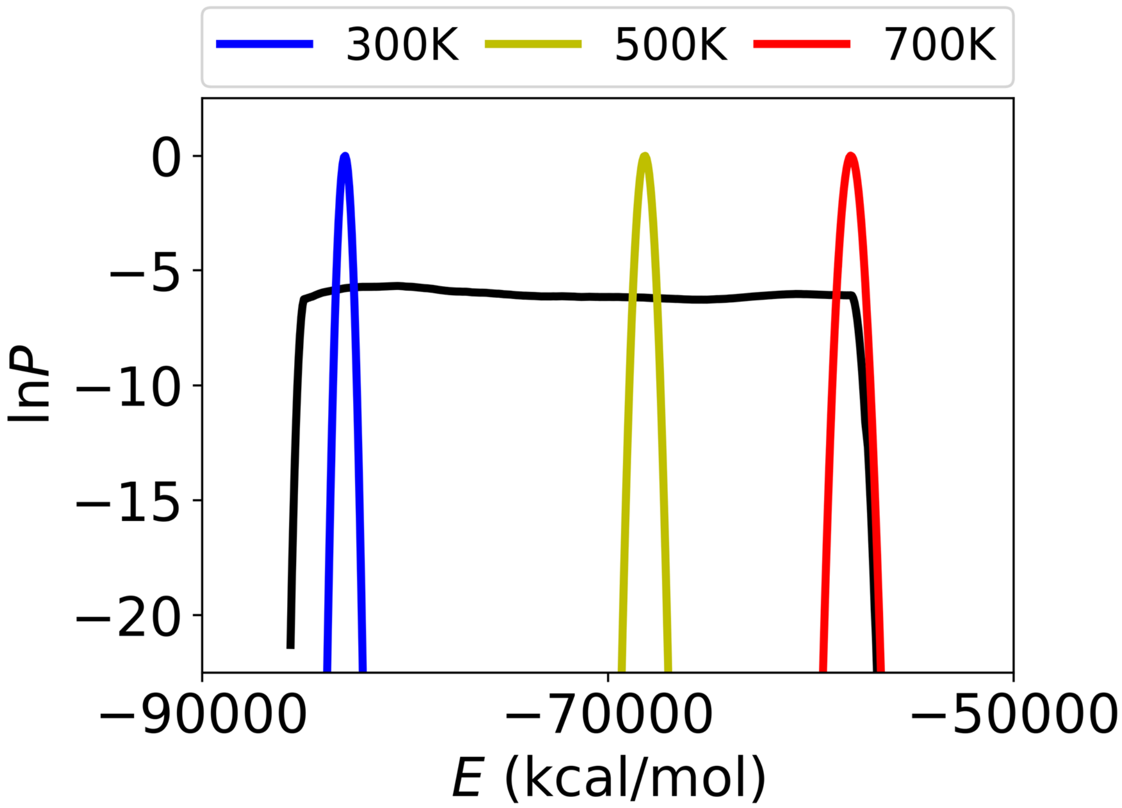

Figure S2. ABT-737 McMD potential energy distribution. Potential energy probability distribution ($P_{McMD}(E)$) as sampled during the production run. Also shown are the reweighing canonical distributions ($P_c(E, T)$) at 300 K, 500 K and 700 K in blue, yellow and red, respectively.

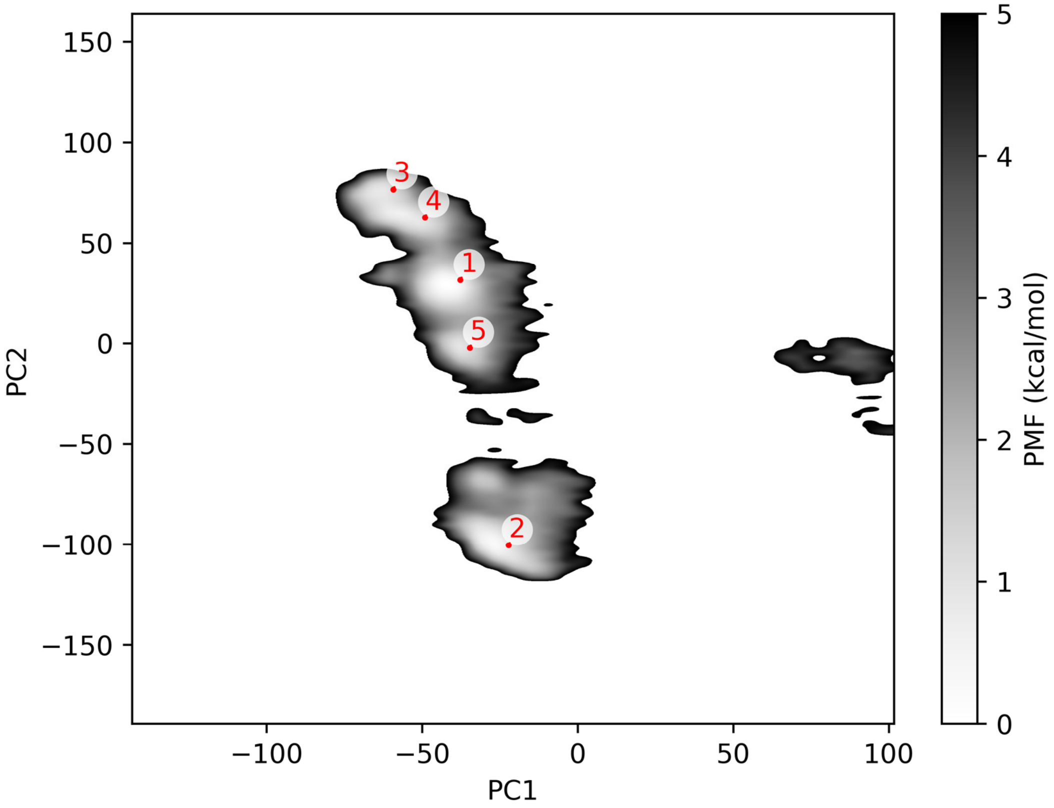

Figure S3. Location of picked representative configurations r_k^W on the FEL.

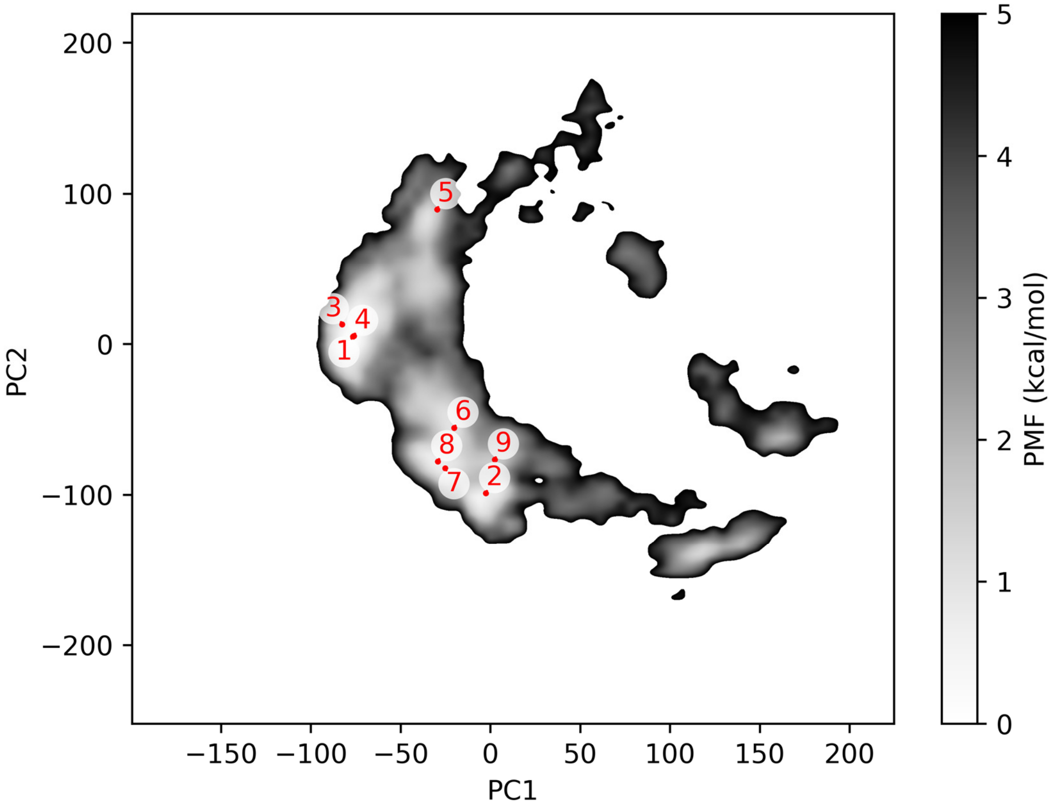

Figure S4. Location of picked representative configurations r_k^A on the FEL.

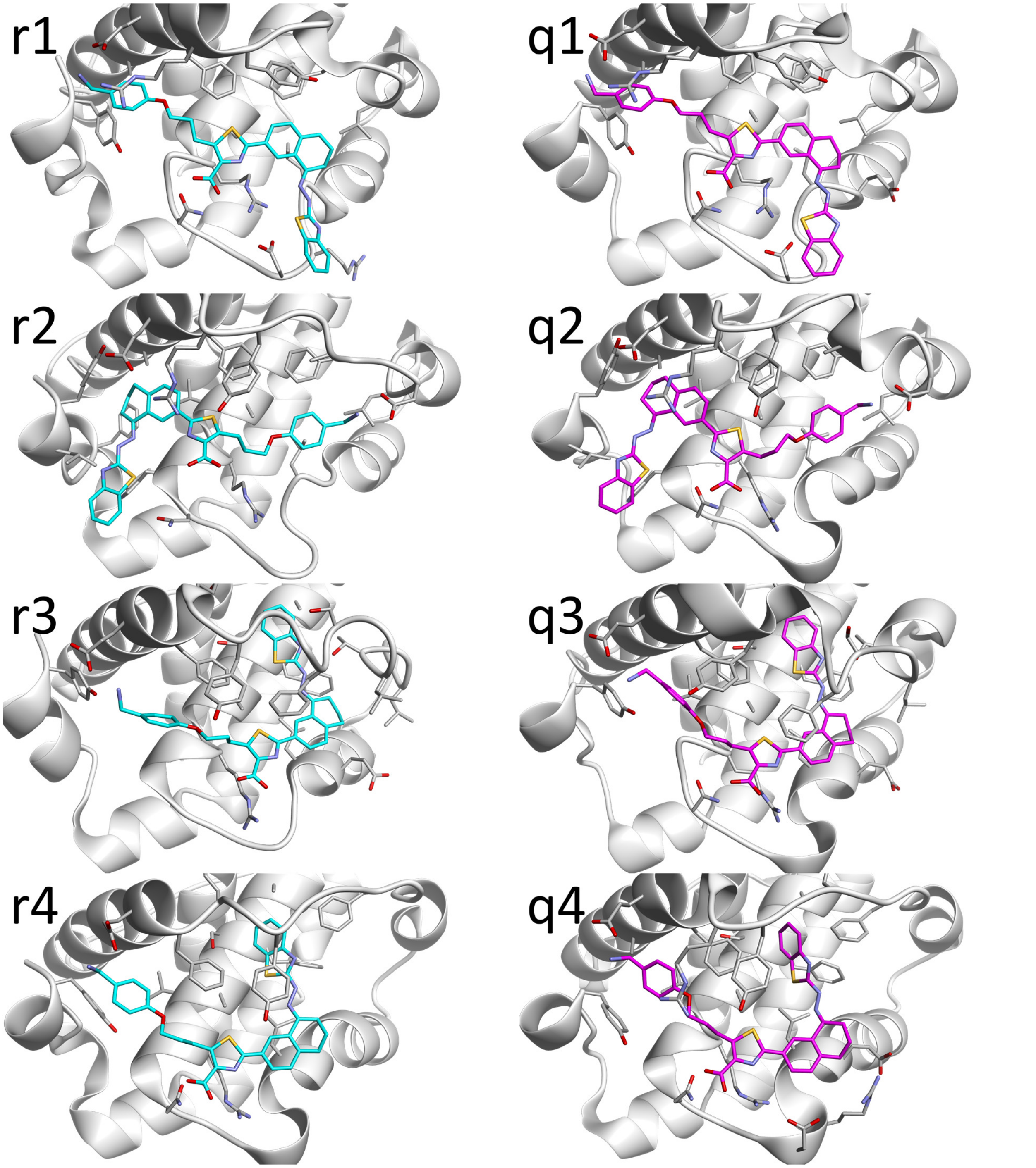

Figure S5. 3D structure of picked representative configurations $\boldsymbol{r}_k^W$. Representative structures from the dynamic docking simulations $\boldsymbol{r}_k^W$ (right) and equilibrated structures $\boldsymbol{q}_k^W$ (left) are shown with the sidechains of the nearby residues shown.

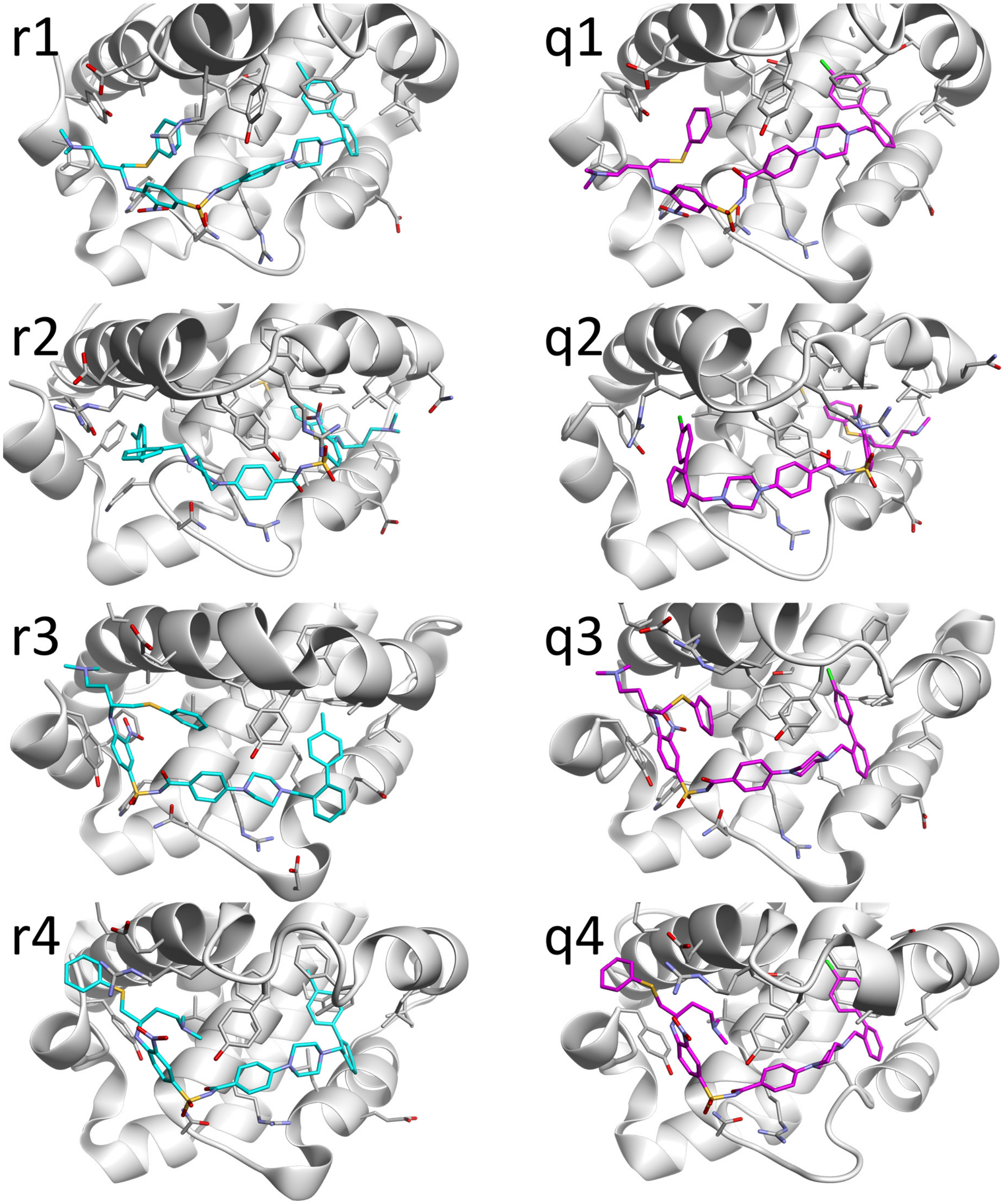

Figure S6. 3D structure of picked representative configurations $\boldsymbol{r}_k^A$. Representative structures from the dynamic docking simulations $\boldsymbol{r}_k^A$ (right) and equilibrated structures $\boldsymbol{q}_k^A$ (left) are shown with the sidechains of the nearby residues shown.

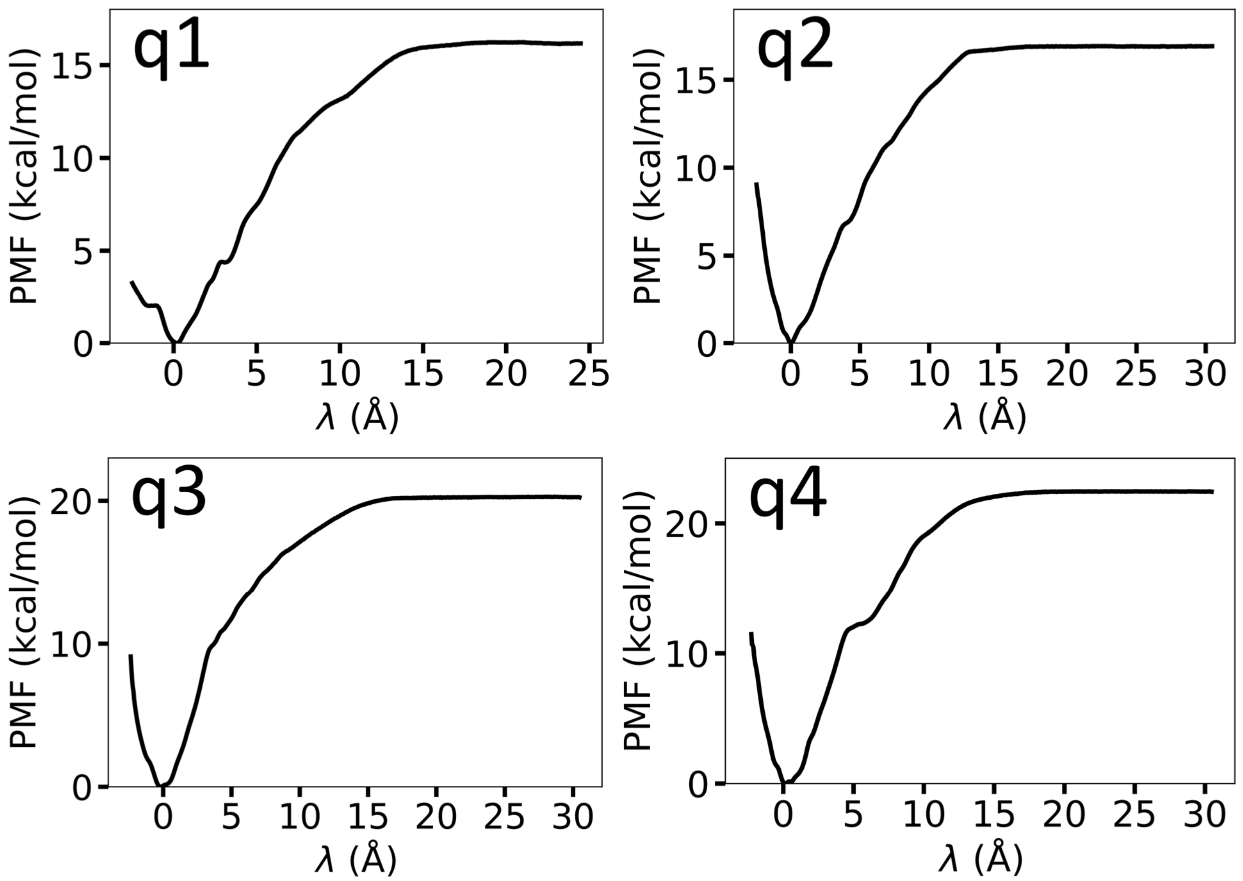

Figure S7. PMF profile for each picked representative configurations q_k^W. For each ligand k=1 through k=4, the potential of mean force (PMF) profile as calculated using WHAM from our path sampling simulations is shown for their respective reaction coordinates (λ).

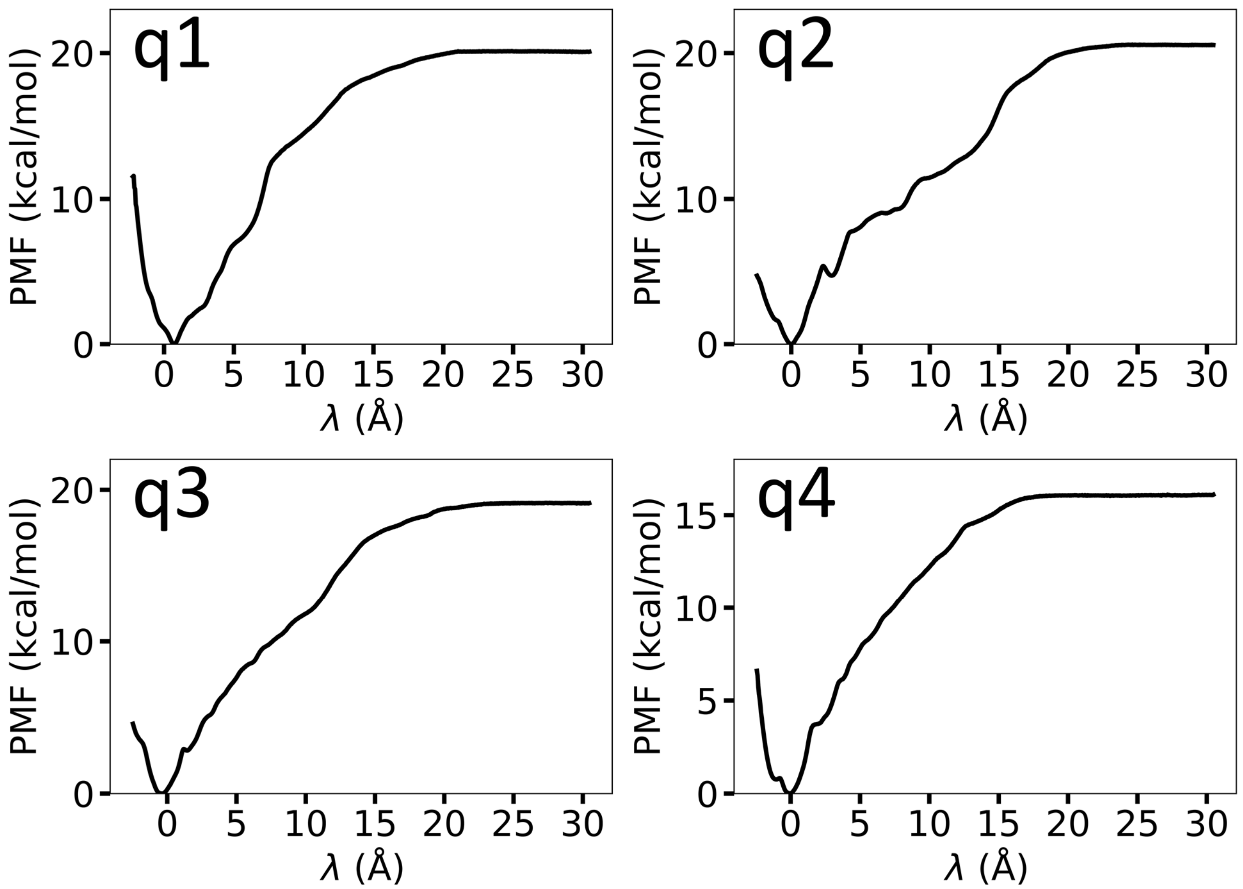

Figure S8. PMF profile for each picked representative configurations q_k^A. For each ligand k=1 through k=4, the potential of mean force (PMF) profile as calculated using WHAM from our path sampling simulations is shown for their respective reaction coordinates (λ).

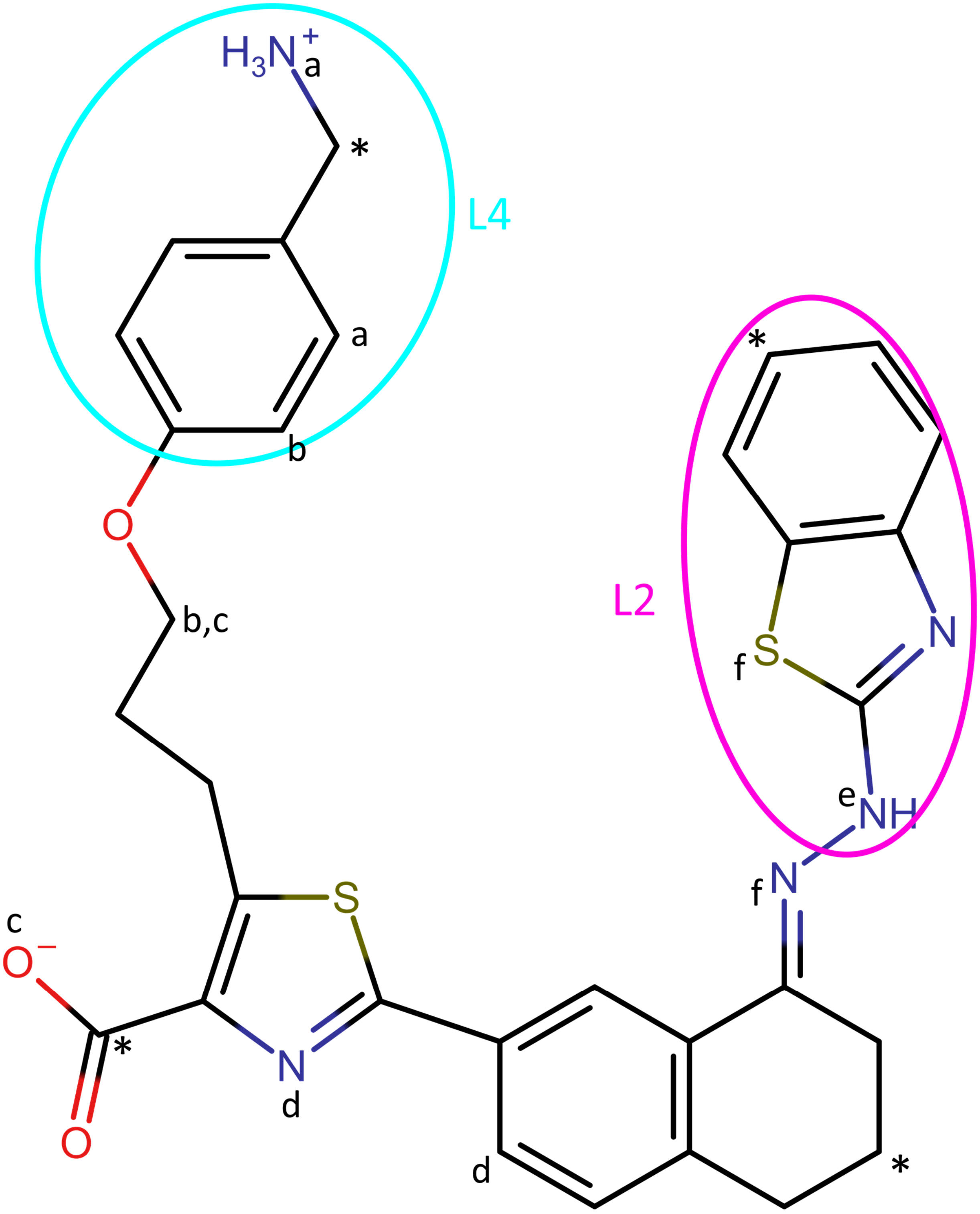

Figure S9. 2D structure of the ligand WEHI-539. Also shown are the atoms used for the PCA, with the inter-ligand pairs a-f and intra-ligand to Bcl-xL Cα atoms indicated by a * symbol. L2 region and L4 region of the ligand is indicated in magenta and cyan, respectively.

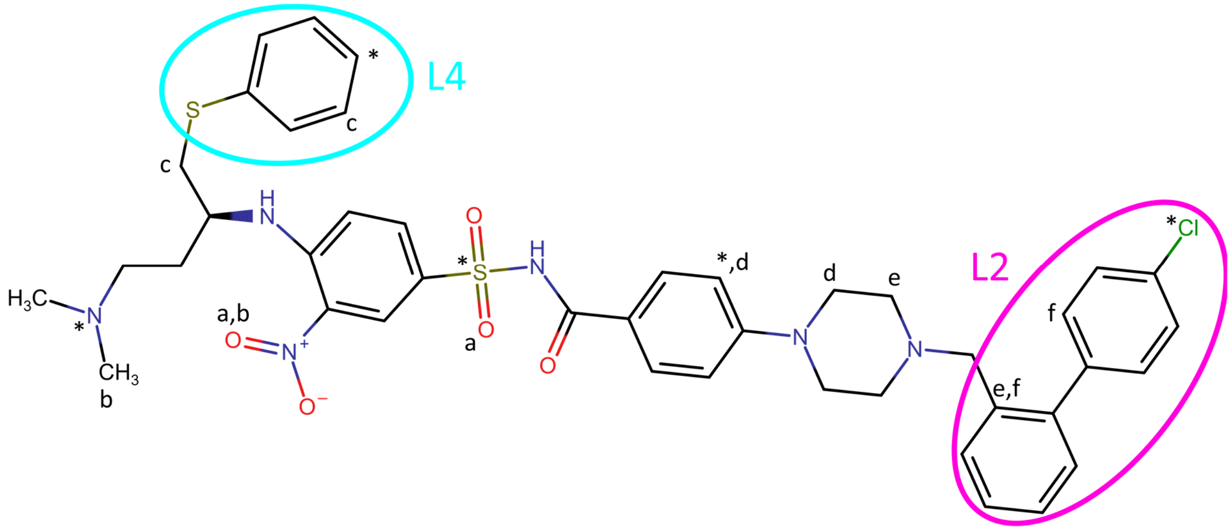

Figure S10. 2D structure of the ligand ABT-737. Also shown are the atoms used for the PCA, with the inter-ligand pairs a-f and intra-ligand to Bcl-xL Cα atoms indicated by a * symbol. L2 region and L4 region of the ligand is indicated in magenta and cyan, respectively.
